# An intelligent agriculture management system for rainfall prediction and fruit health monitoring

**DOI:** 10.1038/s41598-023-49186-y

**Published:** 2024-01-04

**Authors:** Dmitrii Kaplun, Surajit Deka, Arunabh Bora, Nupur Choudhury, Jyotishman Basistha, Bhaswadeep Purkayastha, Ifthikaruz Zaman Mazumder, Vyacheslav Gulvanskii, Kandarpa Kumar Sarma, Debashis Dev Misra

**Affiliations:** 1 Artificial Intelligence Research Institute, China University of Mining and Technology, Xuzhou, 221116 China; 2 Mobile Information Systems Laboratory, Saint Petersburg Electrotechnical University “LETI”, St. Petersburg, 197022 Russia; 3 Department of Electronics and Communication Engineering, Gauhati University, Guwahati, Assam 781014 India; 4 Department of Automation and Control Processes, Saint Petersburg Electrotechnical University “LETI”, St. Petersburg, 197022 Russia; 5 Department of Computer Science and Engineering, Assam Downtown University, Guwahati, Assam 781026 India

**Keywords:** Plant ecology, Software, Computer science

## Abstract

Contrary to popular belief, agriculture is becoming more data-driven with artificial intelligence and Internet-of-Things (IoT) playing crucial roles. In this paper, the integrated processing executed by various sensors combined as an IoT pack and driving an intelligent agriculture management system designed for rainfall prediction and fruit health monitoring have been included. The proposed system based on an AI aided model makes use of a Convolutional Neural Network (CNN) with long short-term memory (LSTM) layer for rainfall prediction and a CNN with SoftMax layer along with a few deep learning pre-trained models for fruit health monitoring. Another model that works as a combined rainfall predictor and fruit health recognizer is designed using a CNN + LSTM and a multi-head self-attention mechanism which proves to be effective. The entire system is cloud resident and available for use through an application.

## Introduction

The world's population is heavily dependent on agriculture for food. Water is a crucial component of agriculture. The yields of crops like fruits, rice, wheat, and other staples with which a sizable portion of the world's population survives depend significantly on the availability and management of water. While 85% of fresh water is used in agriculture, such a vital gift of the nature is becoming increasingly scarce^1,2^. A sizable population depends on agriculture in densely populated nations like India and China, where it generates the majority of the country's income^3^. Contrary to popular belief, the agriculture sector is developing and becoming more data-driven, precise, and intelligent than ever. The rapid developments of Artificial Intelligence (AI) and Internet-of-Things (IoT)-based technology have shifted from statistical to quantitative methodologies in virtually every industry, including "smart agriculture". Such drastic changes are fundamentally altering conventional farming methods and creating new opportunities alongside a number of challenges. Furthermore, there are enough opportunities to develop mechanisms for creating intelligent decision support and process control systems for better plant and fruit health monitoring, automatization of water sprinkling, resource conservation etc. In particular, it is important to investigate performance gains that can be derived by implementing popular, robust and optimized DL networks along with sensors, computer vision and regression techniques and IoT setups in an integrated mechanism.

Given the foregoing, significant efforts have been made throughout the years to utilize modern advancements in AI and IoT to increase the productivity and efficiency of the agriculture sector^4–6^. In recent years, IoT, sensors, and machine learning (ML) / deep learning (DL) have been utilized extensively in the agriculture sector, particularly with regard to monitoring, process control, and management^5,6^. In order to create trustworthy decision support systems (DSS), IoT and ML merge sensor inputs^7,8^. These techniques have made it possible to use data-driven strategies to boost agricultural produce yields. In order to create robust DSS, ML platforms use data collected by sensors as part of IoT setups^9,10^. In particular, ML techniques combine historical facts with the current environment to produce precise forecasts and generate crucial process control decisions^11^. Both wired and wireless sensors are used in agriculture for a variety of tasks, including weather monitoring, self-watering, and other related tasks including monitoring the health of plants and fruits^12–14^. Such prototype hardware-software frameworks enable the fusing of the relevant technologies through which we develop better understanding regarding the assimilation of sensors, data collection and analysis, the creation of a testing environments for sensor fusion, and decision making^15–17^. Such a system designed for agricultural applications must successfully combine sensors in IoT packs and AI aids so as to increase efficiency ^18–24^. Developing a fusion of sensors for designing an agriculture management system for fruit cultivation presents a number of difficulties, including:

- Interface of sensors and devices or hardware components together into a common platform.
- Transmit and receive real-time information through Wi-Fi modules and obtain data analysis.
- Implement ML/DL algorithms for decision support including rainfall prediction and fruit health recognition.
- Control and monitor the integrated management system in real time.

The present work focuses on the design of a precision agriculture setup with stress on the adoption of sensors, IoT, and ML to regulate water flow and process management so as to maximize crop yield. The description included here covers the design of blocks and sub-blocks related to methods for obtaining real-time data from different field sensors, displaying and providing analysis using an Android application, irrigation process control through an automated mechanism, rainfall prediction, and fruit health monitoring using certain ML techniques. A range of sensors, such as soil moisture sensors, temperature and humidity sensors, and air quality sensors, are combined as an IoT set-up. It drives an AI aided decision support system that enables the capture and monitoring of fruit health and the environment of the cultivation ground. Further, the system has the ability to utilize the decisions derived by the AI aided set-up for the control of an irrigation system which is used as part of an agriculture management arrangement. Sensors are synchronized using a microcontroller embedded system, and each dataset obtained from the sensors is sent wirelessly by an IoT device controller to a cloud resident AI tool configured for decision support and monitoring using an Android application. The key aspect of the work is the design and configuration of several ML algorithms, namely CatBoost, Gaussian NB, Random Forest, and Convolutional Neural Network (CNN) with long short-term memory (LSTM) layer for rainfall prediction and fruit health monitoring and recognition using CNN with soft-max layer, CNN with Adam Optimizer and PTM based DL classifiers. These provide area under the curve (AUC) scores as well as accuracy values under field conditions, and provide sufficient ground for the formulation of an integrated agriculture assistant for cultivation and monitoring of certain types of fruits. Further, we have designed a combined rainfall predictor and fruit health recognizer using a CNN + LSTM and a multi-head self-attention mechanism (Section “Combined CNN+ LSTM +attention layer based rainfall prediction and fruit health recognition system”) which has lesser memory requirement, is more accurate and requires low computation time. Experimental results show that the approach is reliable and robust, and the services are hosted using a web portal with a user-friendly interface.

## Related works

A number of technologies have already been reported for the effective use of water using a range of methods and application of precisions aids in agriculture. A system reported in^1^ comprises a distributed wireless sensor network with temperature and soil moisture sensors installed in crop fields. The Zigbee protocol is used to manage sensor data as part of an automated drip irrigation system^2^. The pH and nitrogen levels in the soil are often checked. By monitoring the amount of ground moisture, the authors in^3^ reported the design of a mechanism that can assist an automated watering system. The temperature and moisture levels of the plants are measured by the sensors for moisture and water content^4^. The moisture sensor provides the Raspberry Pi to turn on the water pump and provide the plant with water if the moisture level is determined to be lower than the required level^5^. An IoT based smart irrigation system has been presented in^6^ which reports that the proposed system can regulate soil moisture levels according to requirements and that users may remotely monitor, operate, and gather data through an online website. A range of sensors are available for use in agriculture^7^. In^8^, the authors report the design of a technology that enables the automated, low-cost management of an indoor farm. For the Indian context^9^, suggests using Multiple Linear Regression (MLR) to estimate rainfall. Mean error and root mean square error (RMSE) techniques are used in the design of a model reported in^10^. The model forecasts the rainfall based on the data after the results are examined to determine the inter-annual variability and tested against the experimental yearly variance of rainfall. Rainfall models^10^, optimal microcontrollers^11^, IoT based control system^12^ and greenhouse monitoring^13^, micro-service frontend^14^, etc. are intricately related to precision farming. Some of the recent work^25–28^ have provided diverse approaches towards fruit-health monitoring and use of IoT and ML approaches for a range of decision making and support applications.

## Theoretical background

Here, we discuss the theoretical aspects of different sensors, ML methods and related systems.

### Sensors

**Figure 1:**
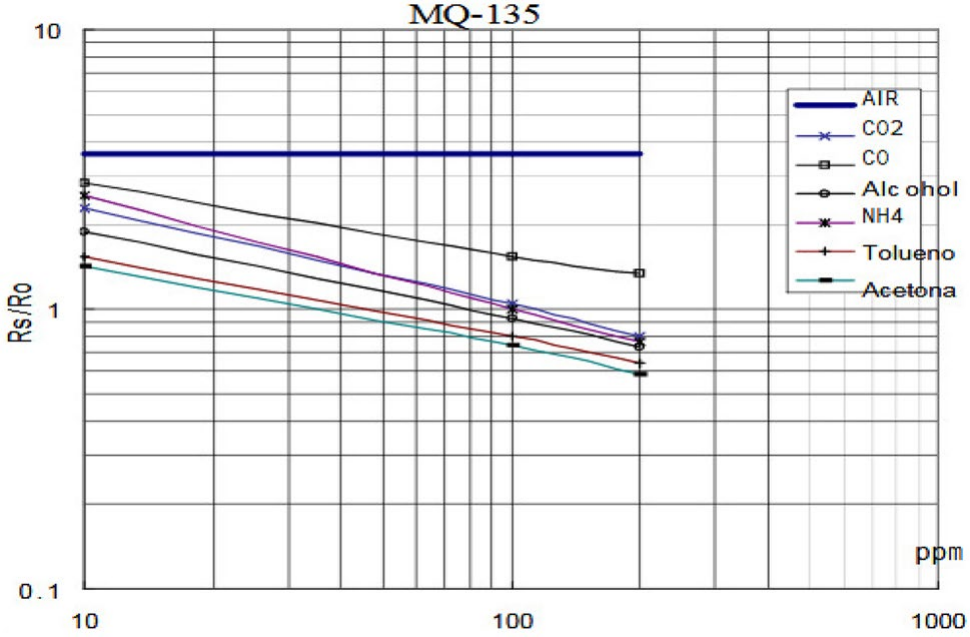
This Sensitivity curve of MQ 135 sensor.

First, we discuss about the sensors. The MQ135 sensor^7,8^ is a low-cost device and suitable for application in areas that deal with air quality and for detecting different types of gasses present in the air. MQ 135 sensor follows a sensitivity curve as shown in Fig. 1. It is a log–log graph and shows plot for multiple gases. The x-axis is the detected concentration of the gas in PPM and the y-axis is the RS/R0 ratio. The equation of a straight line in two-point form is

1 $$y-{y}_{1}=\frac{{y}_{2}-{y}_{1}}{{x}_{2}-{x}_{1}}\left(x-{x}_{1}\right)$$

where (x_1_, y_1_) are the co-ordinates of the first point and (x_2_, y_2_) are the co-ordinates of the second point in the rectangular co-ordinate plane.

Let, y_1_ = 1.122, x_1_ = 10, y_2_ = 0.447 and x_2_ = 100. Therefore, from the sensitivity curve of MQ 135 sensor for acetone, the concentration in PPM for acetone is

2 $${PPM}_{acetone}=159.6-133.33\frac{{R}_{S}}{{R}_{0}}$$

where R_0_ is the resistance of the sensor in clean air and RS is the resistance of the sensor in a gas (acetone).

Another sensor used is the DHT11 sensor. It is used to obtain the temperature and humidity values^8,9^ as our proposed system is designed to work in hot and humid conditions. The soil moisture sensor helps in moisture sensing by measuring the water content in the soil. The sensor has advantages like anti-rusting property and has a long power life.

### Convolutional neural networks (CNNs)

Convolutional Neural Networks (CNNs) are a class of DL models specifically designed for processing structured grid data, such as images and videos. These are inspired by the processing approach of the mammalian cortex^21^. They have revolutionized the field of computer vision and are widely used for various tasks, including image classification, object detection, image generation, and more. Here's a brief overview of CNNs and their advantages:

#### Architecture

- Convolutional Layers: CNNs use convolutional layers that apply learnable filters to input data, allowing them to automatically extract hierarchical features from the input images^21^.
- Pooling Layers: These layers down sample feature maps, reducing the spatial dimensions while retaining important information^21^.
- Fully Connected Layers: After feature extraction, CNNs often include fully connected layers for making predictions or decisions based on the learned features^21^.

#### Advantages of CNNs

- Hierarchical Feature Learning: CNNs automatically learn features at different levels of abstraction. Lower layers capture basic features like edges and textures, while higher layers capture complex patterns and objects. This hierarchical feature learning is crucial for tasks like object recognition^21^.
- Spatial Hierarchy: CNNs respect the spatial hierarchy of data. Convolutional and pooling layers maintain the spatial relationships within an image, which is essential for tasks like object localization^21^.
- Parameter Sharing: CNNs use weight sharing through convolutional kernels. This reduces the number of parameters in the network, making it computationally efficient and reducing the risk of overfitting, especially when dealing with limited training data.
- Translation Invariance: CNNs are naturally invariant to translations in the input data. This means they can recognize patterns or objects in different parts of the image, making them robust to position variations.
- Adaptability: CNNs can be adapted for various tasks with minor architectural changes. Transfer learning allows pre-trained CNNs (e.g., on ImageNet) to be fine-tuned for new tasks, saving training time and data requirements.
- State-of-the-Art Performance: CNNs have achieved state-of-the-art performance in numerous computer vision tasks, including image classification, object detection, facial recognition, and more. They have been applied successfully in diverse domains, including healthcare, autonomous driving, and entertainment.
- Hardware Acceleration: There are specialized hardware accelerators, like GPUs and TPUs, optimized for training and deploying CNNs, making them practical for real-world applications.

### Algorithms for rainfall prediction

**Figure 2:**
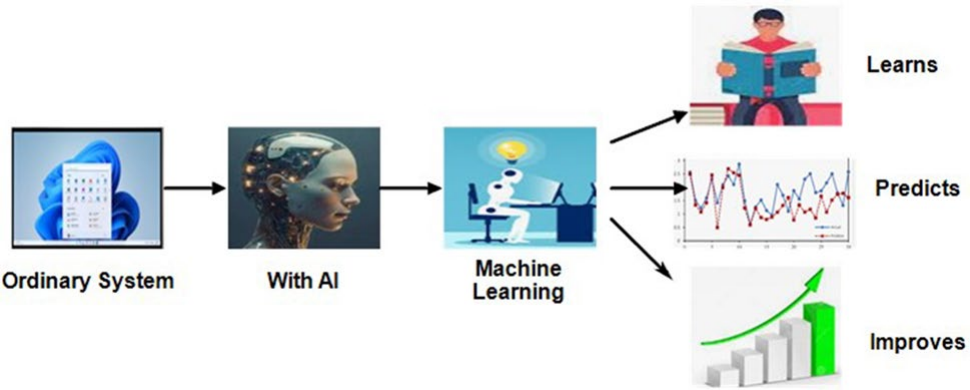
A typical machine learning process.

ML/DL approaches are robust, noise tolerant, adaptive and provide automated decision support (Fig. 2). A few selected ML algorithms are configured for the present task in view of the fast nature of processing and low demands for computational resources.

In the subsequent subsections, all of the ML algorithms that are used in the work configured to carry out the tasks of rainfall prediction (CatBoost classifier, Random Forest classifier, Gaussian NB classifier, CNN + LSTM) are briefly discussed.

#### CatBoost classifier

**Figure 3:**
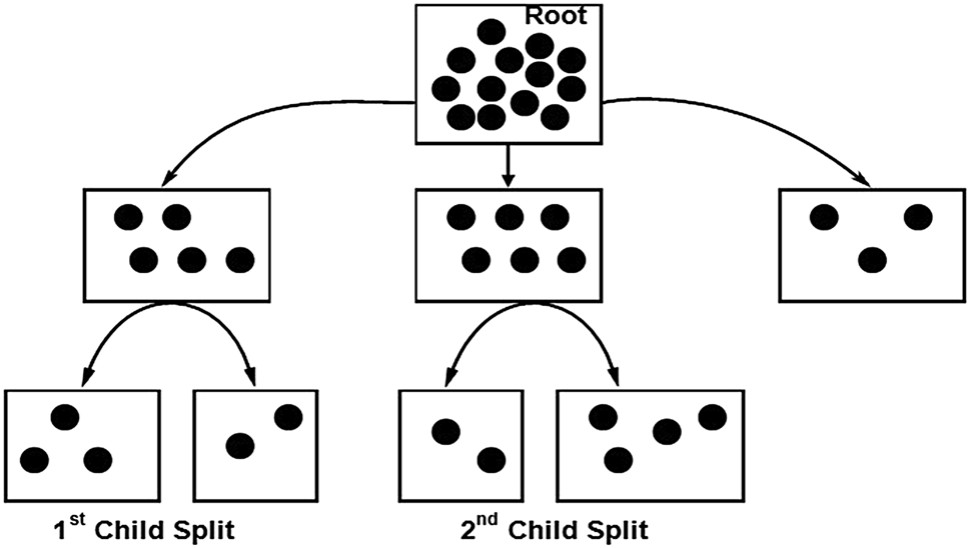
Architecture of CatBoost Classifier.

CatBoost, commonly known as categorical boosting, is a free and open-source boosting toolkit used for a variety of tasks, including regression and classification. Figure 3 displays the various applications of boosted trees for working with categorical variables^14,15,21^. Unlike other gradient boosting methods (which require numeric input), Cat-boost may handle category features. Cat-Boost uses ordered statistics about the goal. As a result, more generic models can be created with less overfitting and less significant hyper parameter adjustments. It's easy to use and works with a wide variety of Python and R packages. CatBoost has been found to have the lowest log-loss value for test data on common ML datasets when compared to other boosting libraries like XGBoost and LightGBM.

#### Random forest classifier

**Figure 4:**
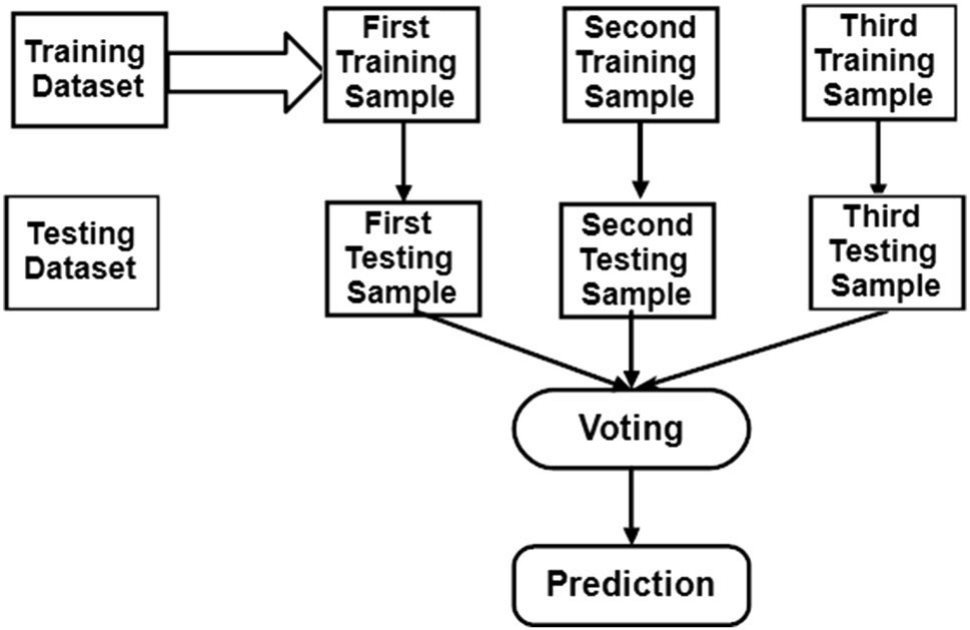
Architecture of Random Forest classifier.

An example of supervised learning may be seen in the Random Forest Classifier (Fig. 4), which can be found in^16,21^. It is applicable to ML problems involving regression as well as classification and can be utilised for both. In order to achieve more accurate results when making predictions based on a specific dataset, the Random Forest Classifier makes use of a number of decision trees that are applied to distinct subsets of the input dataset. As the number of trees in the forest grows, there is a corresponding reduction in the amount of overfitting that occurs. When compared to other algorithms, its training time is significantly less^16^. Mean square error (MSE) is essential for performing regression and it is expressed as

3 $$\mathrm{MSE}=\frac{1}{\mathrm{N}}\sum_{\mathrm{i}=1}^{\mathrm{N}}\left[{\left({\mathrm{f}}_{\mathrm{i}}-{\mathrm{y}}_{\mathrm{i}}\right)}^{2}\right]$$

where, N is the number of all the data points, f_i_ is the value returned by the model and y_i_ is the model returned by the actual data points i. For classification-based problems, the standard expression used is

4 $${\mathrm{G}}_{\mathrm{ini}}=1-\sum_{\mathrm{i}=1}^{\mathrm{C}}\left[{\left({\mathrm{P}}_{\mathrm{i}}\right)}^{2}\right]$$

G_ini_ index is calculated by subtracting the sum of the squared probabilities of each class from one.

#### Gaussian naive bayes (NB) classifier

A unique variety of NB algorithm is the Gaussian Naive Bayes algorithm^21^. When the dimensionality is high, it has a high level of utility. Even with little training data, it can function^17^ effectively. The Gaussian's equation for NB

5 $${\mathrm{P}}\left({\mathrm{x}}_{\mathrm{i}}|{\mathrm{y}}\right)=\frac{1}{\sqrt{2{\pi} {\upsigma }_{\mathrm{y}}^{2}}}{\mathrm{exp}}\left[\frac{-{\left({\mathrm{x}}_{\mathrm{i}}-{\upmu }_{\mathrm{y}}\right)}^{2}}{2{\upsigma }_{\mathrm{y}}^{2}}\right]$$

where P is the Probability function, σ_y_^2^ is the variance and µ_y_ is the mean of the Gaussian random variable y.

**Figure 5:**
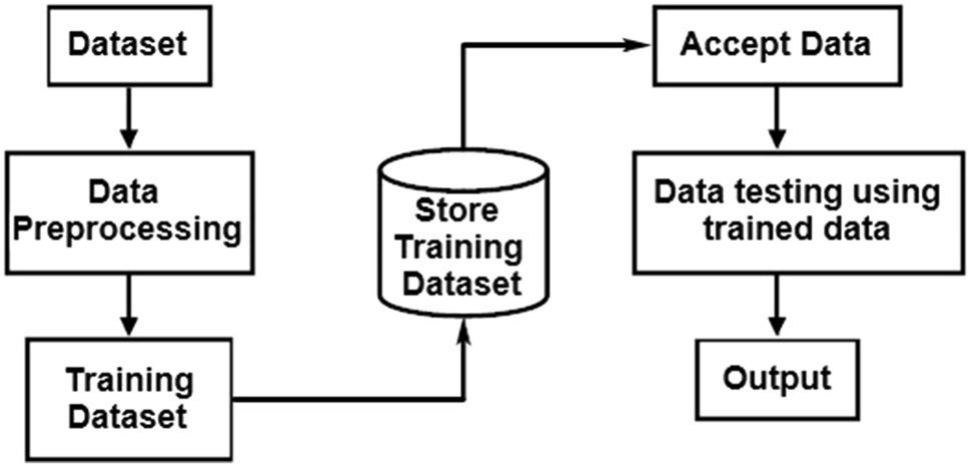
Architecture of Gaussian NB classifier.

Architecture of Gaussian NB classifier is presented in Fig. 5.

#### CNN + LSTM-based classifier

**Figure 6:**
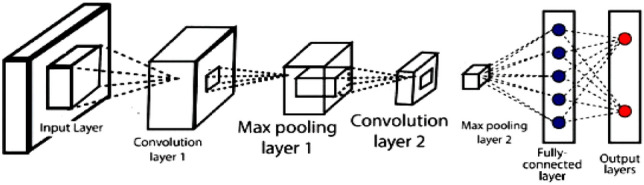
Architecture of CNN used for fruit health monitoring.

In particular, it is important to investigate performance gains that can be achieved by implementing popular DL networks along with computer vision techniques and IoT setups in an integrated strategy. Better performance, especially with real-time inputs, is anticipated for DL structures that can handle spatial and temporal changes in samples. The spatial information of samples, especially those from visual inputs, can be reliably captured by CNN-based structures, but features with time-varying characteristics are difficult to handle^21^. This is especially pertinent with rainfall prediction. Recurrent structures like the LSTM cells are thought to be effective for temporal properties^21^. Additionally, integrated and AI-assisted frameworks with the capacity to extract spatial and temporal variations when used for rainfall prediction on continuously captured on-field sensor data have relevance not only for the farming community but also for ecological conservation. Hence, we have used a hybrid structure of CNN (Fig. 6) and LSTM for rainfall prediction using sensor fed data.

### Algorithms for fruit health recognition

In this subsection, all of the ML techniques are discussed that are used in the fruit health recognition model. For fruit health monitoring and recognition, we have used CNN + Softmax, Adam Optimizer, ResNet50 and GoogleNet.

#### CNN with softmax classifier layer

Deep learning neural networks like CNN (Fig. 6), are frequently used to interpret structured arrays especially while capturing patterns from input images^21^. CNN contains several layers, each of which is capable of recognising intricate forms and details. Here, maxpooling 2D and Conv 2D are used. A two-dimensional filter is slid over each channel during a maxpooling process, covering any features that are present there. Conv 2D is a two-dimensional convolutional layer which helps in creating a kernel that is wind with layer input which helps to produce tensor of output, in other words, the number of filters that convolutional layers will learn from^18^. At the end, the CNN is terminated by a softmax layer which performs the classification.

#### Adam optimizer with CNN

Adam Optimizer, often referred to as Adaptive Moment Estimation, is a method for gradient descent. It is a very useful and efficient algorithm that uses minimal memory. It essentially mixes Gradient Descent with Momentum and the Root Mean Square Propagation (RMSP) technique^18^. The gradient descent algorithm is accelerated by the momentum algorithm using the gradients' exponentially weighted average. RMSP, which employs the exponential moving average, improves AdaGrad. The two methods discussed above are combined by Adam Optimizer to inherit the advantages and provide a more potent optimised gradient descent when combined with CNN.

#### PTMs

We have also used PTMs ResNet50 and GoogleNet^21^ for fruit health recognition which provide better insight regarding the efficacy of DL based structures for design of smart agriculture assistant.

## Methodology

In this section, we discuss the methodology of the design of the system. The description includes the design of the integrated IoT system, deal time database design, development of an Android app, design of rainfall prediction and fruit health monitoring systems, full-stack web development and the formulation of a combined CNN + LSTM + Attention Layer based rainfall prediction and fruit health recognition system. Each of the blocks are designed separately, tested and integrated. Further, the integrated system is validated and tested extensively to determine the performance limits. In the following sub-sections, each of the blocks are discussed with all relevant details.

### Design of an integrated IoT system

**Figure 7:**
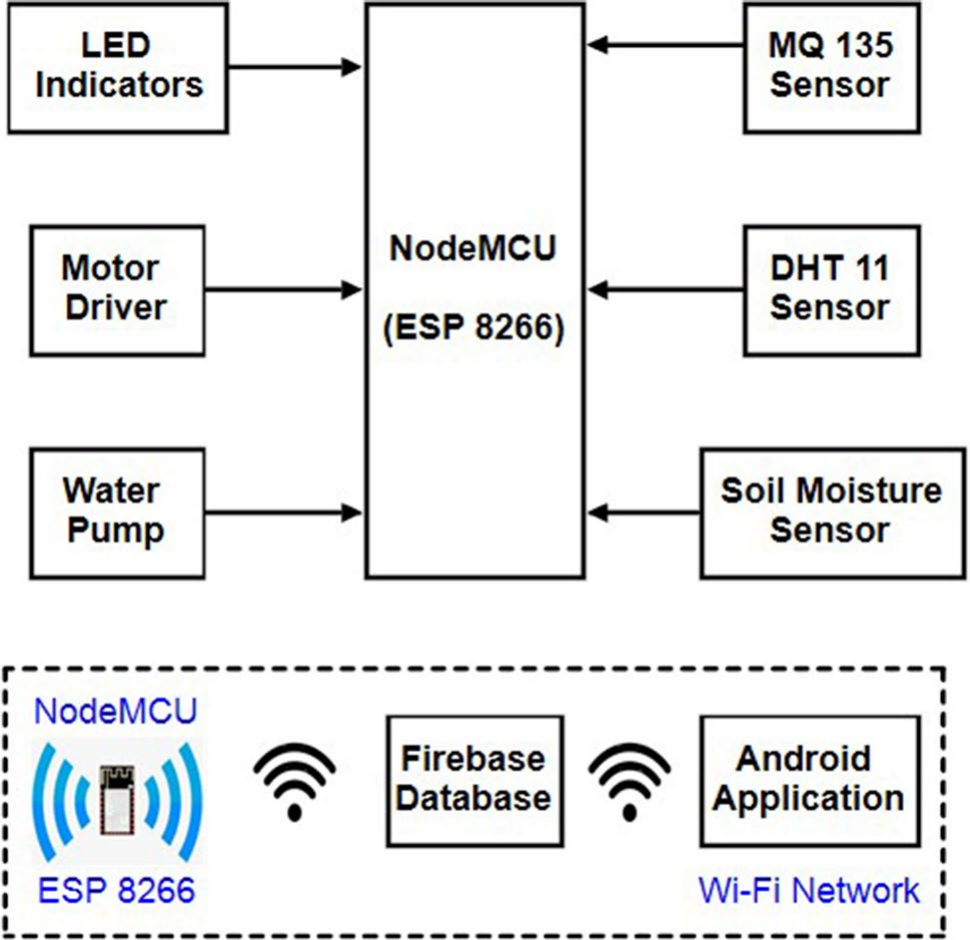
Block diagram of the proposed IoT system.

Figure 7 depicts the system model, which includes both manual control of the system via a mobile device and the use of various sensors including decision support as part of an automated farming assistance system. NodeMCU, various sensors, including soil moisture sensors, temperature and humidity sensors, and air quality sensors, LEDs, DC water pumps, and motor drivers make up the hardware. For data collection, sensors are inserted into a variety of locations in the field. The soil moisture sensor determines the volumetric water content of the soil by using some of the soil's properties, such as electrical resistance and dielectric constants. Two probes are put into the soil to make up the device. The temperature and humidity sensor (DHT11) are a multipurpose sensor that simultaneously reports the temperature and relative humidity. The air quality sensor (MQ 135) measures air quality in parts per million (PPM), making it perfect for detecting NH_3_, NOx, alcohol, benzene, smoking, CO_2_, and other substances. Organic solvent acetone has an impact on soil processes. Different agricultural and ornamental plants are adversely affected by acetone, which also reduces their size and damages their membranes. NodeMCU gathers real-time environmental inputs from various sensors and combine it, improving the system's data logging and aiding the AI based automation strategy. The farmer needs the access to real-time data collected by various sensors and shall be able to take control of the motors as needed using the android application. LEDs are used to show whether the sensors are functioning. A Red LED placed close to the sensor will flash to show the system is malfunctioning if a certain sensor cannot read data or if there is a connectivity problem. Additionally, we can determine the sensor's location based on where the LED illuminates.

**Figure 8:**
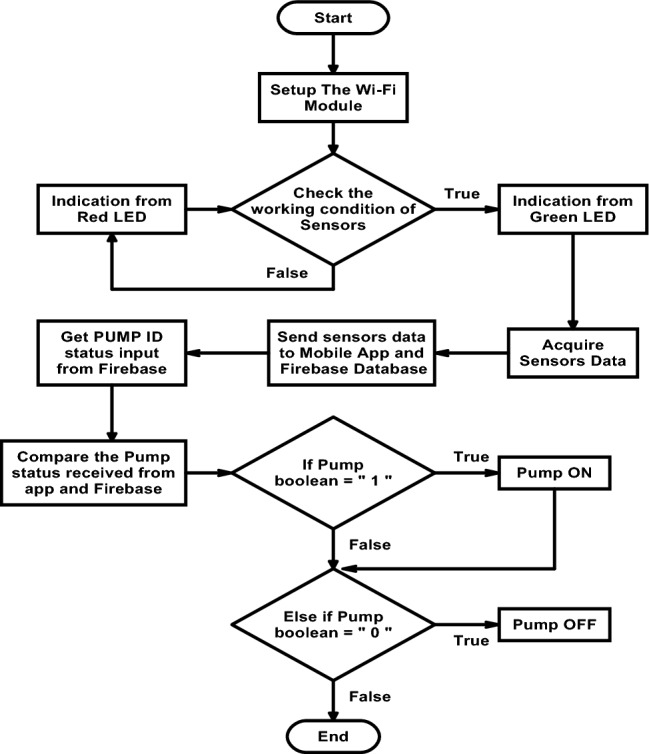
Flow chart of the proposed IoT system.

Figure 8 depicts the proposed system's flowchart along with the necessary combinations of various blocks. The WiFi module is first set up during development, and then our program tests to see if the sensors are working properly. If there is a connection issue or if the sensor is unable to read the data, the Red LED will indicate. Whether or not everything is functional will be shown by the green LED. The sensors' data will subsequently be gathered and transmitted to the mobile app and Firebase database. The pump status that the program obtains from the app and database can be compared. If the observed pump status is 1, the pump will be on, and if it is 0, the pump will be off. Algorithm 1 summarizes the process logic.

**Algorithm 1:**
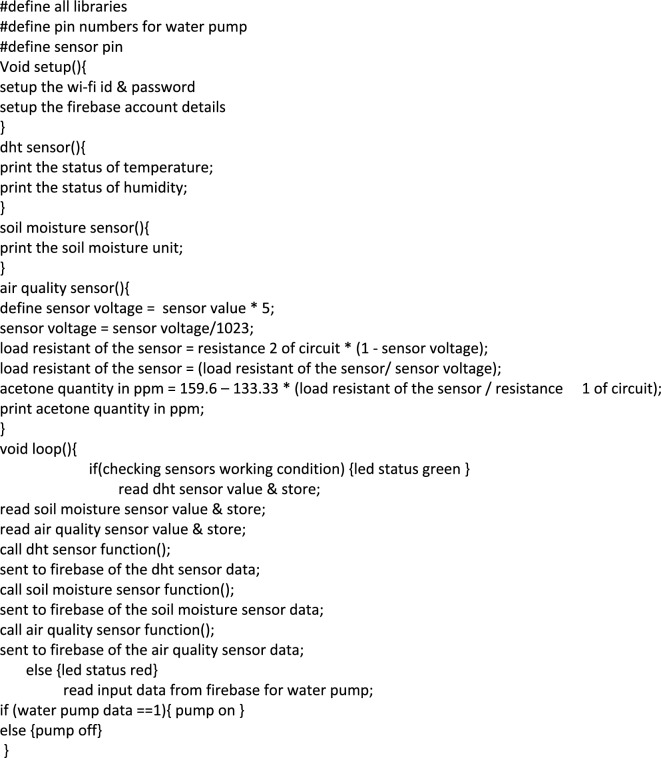
Working of the proposed approach

### Real-time database designing

**Figure 9:**
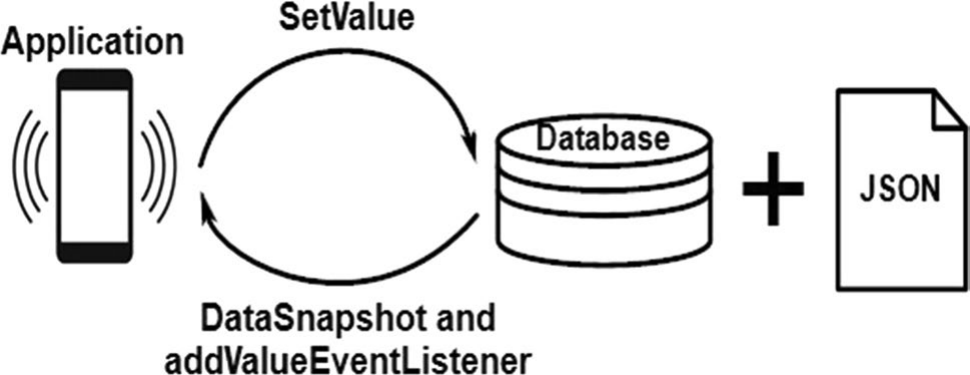
Firebase read and write operations through the Android apk.

**Figure 10:**
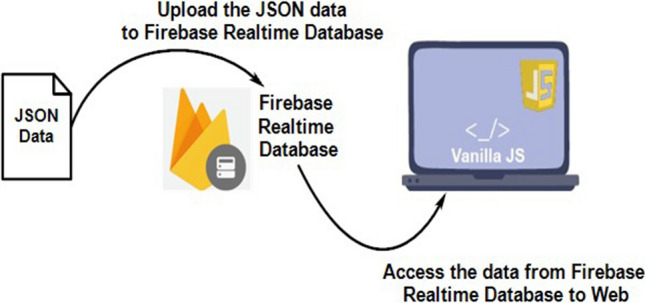
Accessing Real-time data from JSON through the web interface.

On the Google Firebase platform, a database is built to collect real-time data (Fig. 9). Every connected client receives a real-time synchronization signal of data that is stored as JSON (Fig. 10). To create a Google Firebase account, users must sign in with their email addresses. Following sign-in, one Firebase account is created in private mode. Before it can be connected, the NodeMCU board must change from private to public mode by altering a property. The second stage is where you can find the fingerprint address of the website. The address should be in the "httpclient.c" file of the NodeMCU library. The Firebase host address on the ESP8266 should be set to the IP address of the Firebase account.

### Development of android app

**Figure 11:**
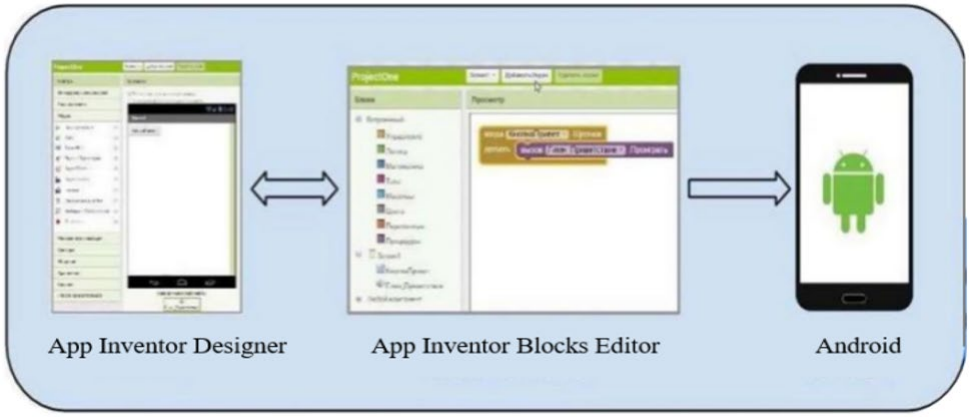
Architecture of the Android app development in MIT App Inventor.

An Android application is developed to monitor the real-time data and control the irrigation system. The Massachusetts Institute of Technology (MIT) now manages MIT App Inventor, a web application integrated development environment that was first made available by Google. This open-source web application is used to establish a real-time data monitoring program (Fig. 11). The Google Firebase platform is accessed by this app using the Firebase IP address and API Key. By entering these criteria in the mobile app development dashboard, all pertinent components (such as buttons, labels, fire-based, etc.) are used in the design of the app interface. When a mobile app is generated, an Android device scans its QR code in the APK file to install it.

### Rainfall prediction

**Figure 12:**
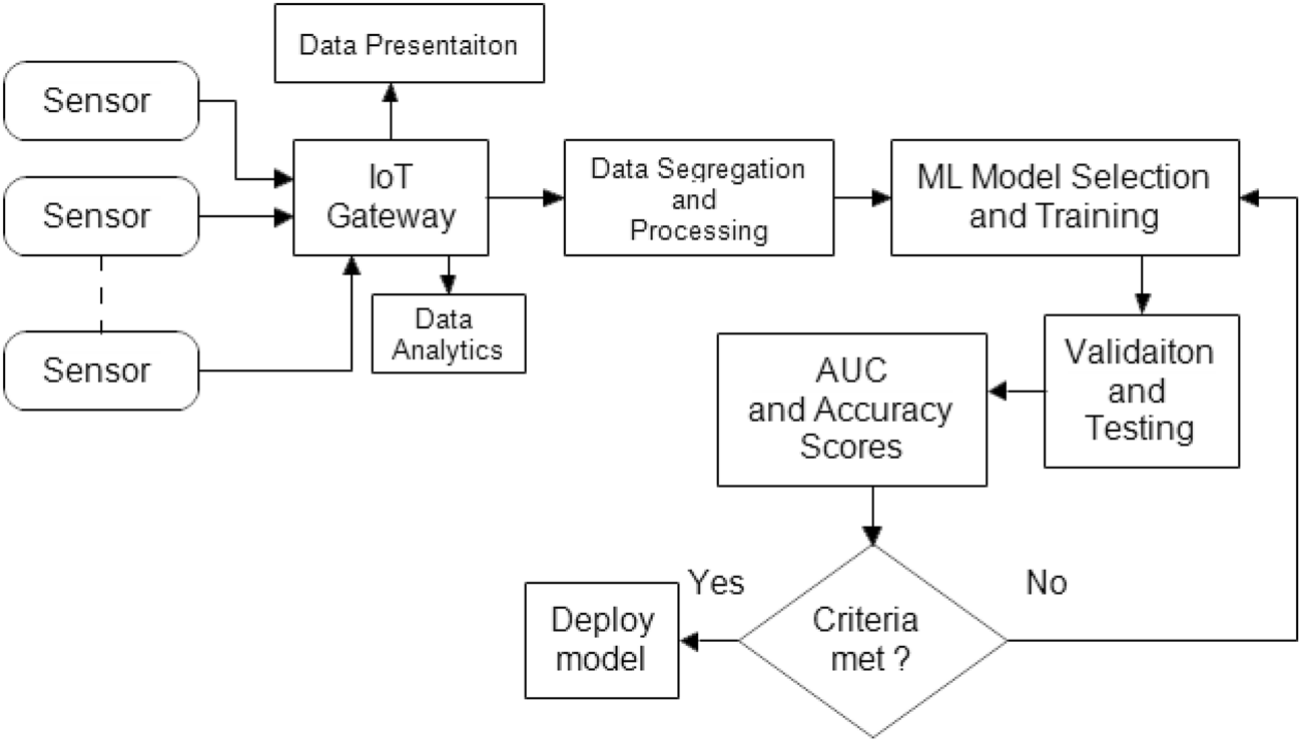
Block diagram of rainfall prediction model.

The goal of the rainfall prediction model (Fig. 12) is to preserve water by using ML to accurately forecast rainfall, prepare for contingencies, manage irrigation facilities and use the rainwater if water supplies are scarce. The forecast for the rainfall is based on a weather dataset from Kaggle. Once the data has been read into Jupyter Notebook, missing values for all categorical and numerical variables are handled using level encoding, median, get dummies, and random imputation. Outliers are also eliminated from the data box plot. The categorical variables "Rain Tomorrow" and "Date" are selected for training and testing after data analysis. Several methods, including CatBoost Classifier, Random Forest Classifier, Gaussian NB, and CNN + LSTM network, are used to calculate the AUC and accuracy score. The trained networks are fed with sensor data to provide relevant real-time responses.

A web resident functionality provides indication of weather condition and rain forecast of a locality. The "Predictor Section," provides the rainfall forecasting. The user can enter several input factors for a certain day that are necessary for determining whether it will rain or not. Date, highest and lowest temperatures, evaporation, sunshine, wind gust speed, humidity, wind speed, pressure, and cloud at specific time are some of the input factors. The user must select the "Predict" option after entering the required parameters (Table 1) in order to forecast the amount of precipitation.

**Table 1:** Parameters used in rainfall prediction model.

Parameters	Significance
Numerical features count = 16	Numerical features are used to represent integers and float
Discrete features count = 2	Discrete features can take only a specific value
Continuous features count = 14	Continuous features have a range of values
Categorical features count = 7	Categorical features can take on levels or values
Cat-boost classifier	Reduces the need for extensive hyper-parameter tuning and lower the chances of over-fitting leading to effective accuracy
Random forest classifier	Capable of handling large datasets with high dimensionality and accuracy
Gaussian NB classifier	Gaussian NB classifier

### Fruit health recognition

**Figure 13:**
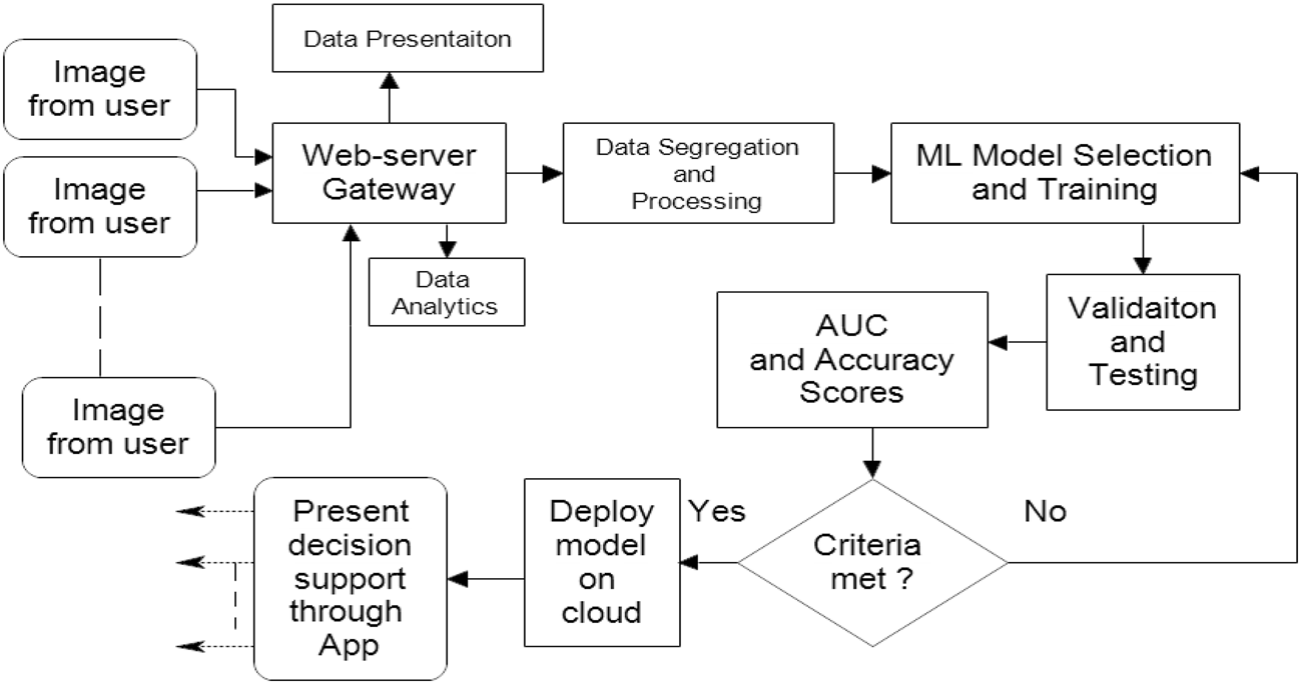
Block diagram of fruit health recognition model.

Fruit health recognition system (Fig. 13) is designed with a view to distinguish fresh fruits from rotten fruits. The overall productivity and profitability would suffer if rotten fruits are stored along with the fresh fruits as the rotten ones will damage the fresh ones. Fruit recognition system is useful in mitigating this issue.

**Figure 14:**
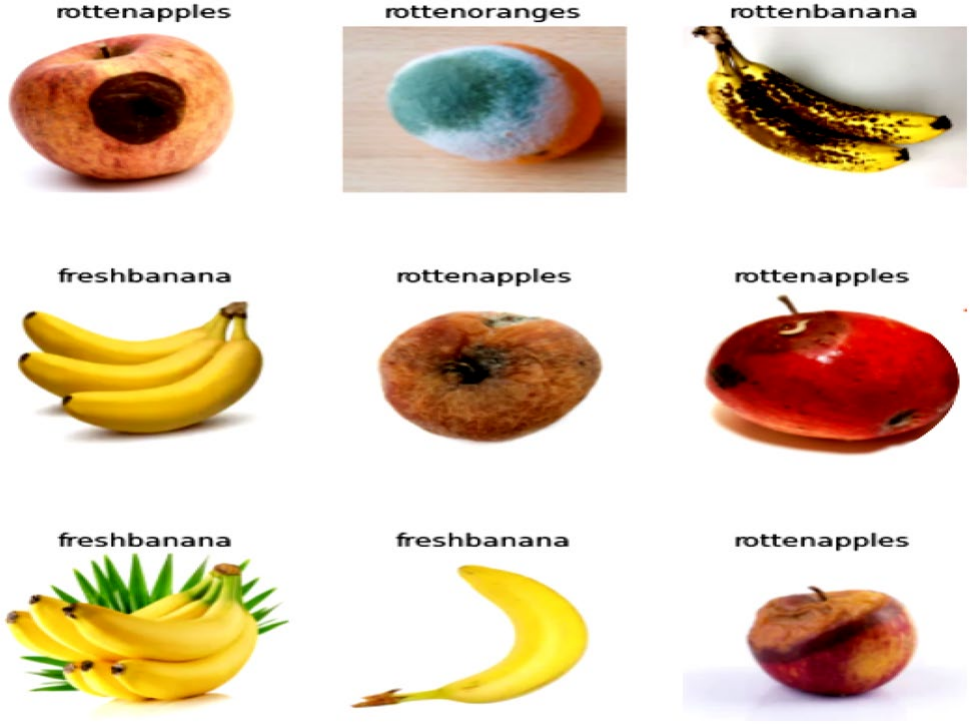
Portion of the sample images taken for training, validation and testing of fruit health recognition model.

Here in this model, three different fruits are used: Bananas, Apples and Oranges. Rotten and fresh images for all three are collected (Fig. 14). For each class there are at least three different varieties in shape and two in colour. The size of the database is 4200 images of which 50% are used for training and the remaining for validation and training. Further, two illumination and three resolution captures are added which augmented the data size significantly.

The paper contains the preprocessing steps involving standardization, normalization, and rescaling of the images regarding the novelty of the work. Normalization is applied here to scale the pixel values of the images to a common range, typically between 0 and 1. This helps ensure that the model trains effectively without being influenced by the varying pixel value scales in different images. It is necessary to establish a benchmark standard for designing and testing the complete system based on AI techniques. One the system is validated, the normalization process is not required. We have calculated the mean and standard deviation of pixel values across the entire dataset and we have used these statistics to normalize each image. The formula for normalization is

6 $${\text{Normalization}} = \left( {{\text{Pixel Value}} - {\text{Mean}}} \right)/{\text{Standard Deviation}}$$

After normalization the pixel values are in a consistent range, reducing the likelihood of vanishing or exploding gradients during training. Also the model can learn faster and generalize better. The impact of rescaling involves resizing the images to a common and manageable size. It's essential for models that require fixed input dimensions, such as CNNs. For the procedure of rescaling we have resized each image to a specified target size, e.g., 300 × 300 pixels. When resizing, we have maintained the aspect ratio to avoid distortion. The benefits of rescaling ensures that all images have the same dimensions, making them compatible with models like CNNs and also reduces computational complexity and memory requirements, especially for deep learning models.

In order to increase the number of images on the dataset, data augmentation on the existing images is performed. In the Jupyter Notebook, various modules such as TensorFlow, Keras, Matplotlib, Pyplot and Seaborn are uploaded. Dataset is divided into training and testing dataset. Maxpooling 2D, Conv 2D and Adam optimizer are implemented as part of the popular CNN structure. Accuracy and validation scores of the model are obtained and a graph is plotted between training and validation accuracy and losses. Table 2 summarizes the different parameters used in the design of the fruit health recognition system. A webpage is deployed that provides an interface to the user for fruit health recognition, from where the prediction regarding the condition of the fruit is done. We have used TensorFlow as the deep learning framework for building and training our fruit health recognition model.

**Table 2:** Parameters used in fruit health recognition model.

Parameters	Significance
TensorFlow	Defines a helper function to demonstrate the errors
Image_Size = 300	All the images are of 300 × 300
Batch_Size = 32	Batches are of 32 sizes
Channels = 3	RGB channels
Epochs = 35, 70, 100, 150, 200, 250	35 number of passes are used
Data size	4500 images augmented with two illumination and three resolutions
Gaussian NB classifier	Gaussian NB classifier
Size of the training dataset	50%
Size of the validation dataset	25%
Size of the test dataset	25%
Trail repetitions and averaging after	5 trials
Data augmentation	Increases the amount of data by generating new data points
Conv2D	Increases the number of filters that convolution layers will learn from
MaxPooling2D	A two-dimensional filter slided over each channel and the features lying within the channel is covered
Adam optimizer	Useful and effective because it uses less memory
verbose = 1	Allows to write expressions that are more readable by visually separating logical sections

The parameter selection of the training and dataset are based on the following considerations:

#### Deep learning framework—TensorFlow

*Image_Size* = *300* × *300* The images in our dataset have been resized to 300 × 300 pixels to fulfill the requirements of training the AI system.

*Batch_Size* = *32* During training, the model processes data in batches, with each batch containing 32 images.

*Channels* = *3 (RGB)* The images in our dataset are in RGB format, with each image having three color channels (Red, Green, and Blue).

*Epochs* = *35, 70, 100, 150, 200, 250, 35 number of passes are used* We have specified a range of epochs for training the model. Each epoch represents one pass through the entire training dataset. It appears that our plan to train the model for different numbers of epochs to assess its performance.

*Data size* = *4500 images augmented with two illuminations and three resolutions* The dataset consists of 4,500 images. Additionally, data augmentation techniques have been applied, including variations in illumination and three different resolutions. Data augmentation is a valuable technique for increasing the diversity of the training data.

*Gaussian NB classifier* However, in the context of deep learning and image recognition, deep neural networks, particularly CNNs, are typically used instead of Gaussian NB. CNNs are better suited for image-based classification tasks^21^.

*Size of the training dataset* = *50%* Half (50%) of the dataset is allocated for training the model. This is a common practice, as it allows the model to learn from a substantial portion of the data.

*Size of the validation dataset* = *25%* A quarter (25%) of the dataset is allocated for validation. The validation dataset is used to assess the model's performance during training and to tune hyper parameters.

With these parameters, we have proceeded with training the fruit health recognition model using TensorFlow. However, the use of CNNs instead of Gaussian NB is necessitated for the requirements of better performance in case fruit health classification tasks. Additionally, monitoring the model's performance on the validation set during training is used to make informed decisions about model architecture and hyper parameter tuning. The parameters used in fruit health recognition model is given in Table 2.

### Full-stack web development

For the proposed work, three full-stack websites are developed that includes all the aspects of the work. Technologies employed for the web development include:

- For Front-End—HTML, CSS, JavaScript etc.
- For Back-End – Flask.

#### Agricultural management system website

The main website is created with the title ‘Agricultural Management System’. A navigation bar is created with different contents from where user can navigate to the different sections of the website like “About”, “Services” that are offered, “Contact” to the Developers etc. The most important section “Services” consists of different features like Weather Forecasting, Fruit Health Recognition, APK for real-time data monitoring and irrigation, online education platform etc. The farming-based online education platform, which is presently under development, requires users to sign up and pay to enroll. The main website is linked to two other websites: Rainfall Prediction and Fruit Recognition. The user will be redirected to the appropriate website when they select the option for weather forecasting or fruit health recognition.

#### Rainfall prediction website

A website is created with the title ‘Rainfall Predictor’ using HTML, CSS (front-end) and Flask (back-end). From the main website, the Rainfall Predictor website can be accessed directly by clicking “Weather Forecasting option”. A navigation bar is created with different contents from where user can navigate to the different sections of the website like “Home”, “About Rainfall Predictor”, “Dashboard”, “Developers” and the “Predictor” section from where the actual prediction is done. The webpage contains a dashboard which provides graphical representation of the parameters. The user can contact to the developers by navigating into the Developer section.

#### Fruit recognition web service

A web service is created with the title ‘Fruit Recognition’ using HTML, CSS (front-end) and Flask (back-end). From the main website, the fruit recognition website can be accessed directly by clicking “Fruit Health”. A navigation bar is created with different contents from where user can navigate to the different sections of the website like “Home”, “About” the website, “Contact” and the “Predictor” section from where the actual prediction is done. The main feature is the ‘Prediction Section’ from where the actual prediction regarding the condition of the fruit is determined. The user in order to identify the condition of the fruit has to go to the prediction section in the navigation bar from where images of the fruits present in the directory are selected and after selecting the images the user has to click the submit button and the result is displayed featuring the condition of the fruits (fresh or rotten) as well as the confidence percentage. The user can also contact to the developers regarding any query or issue by switching to the “Contact” section in the navigation bar.

### Combined CNN + LSTM + attention layer based rainfall prediction and fruit health recognition system

**Figure 15:**
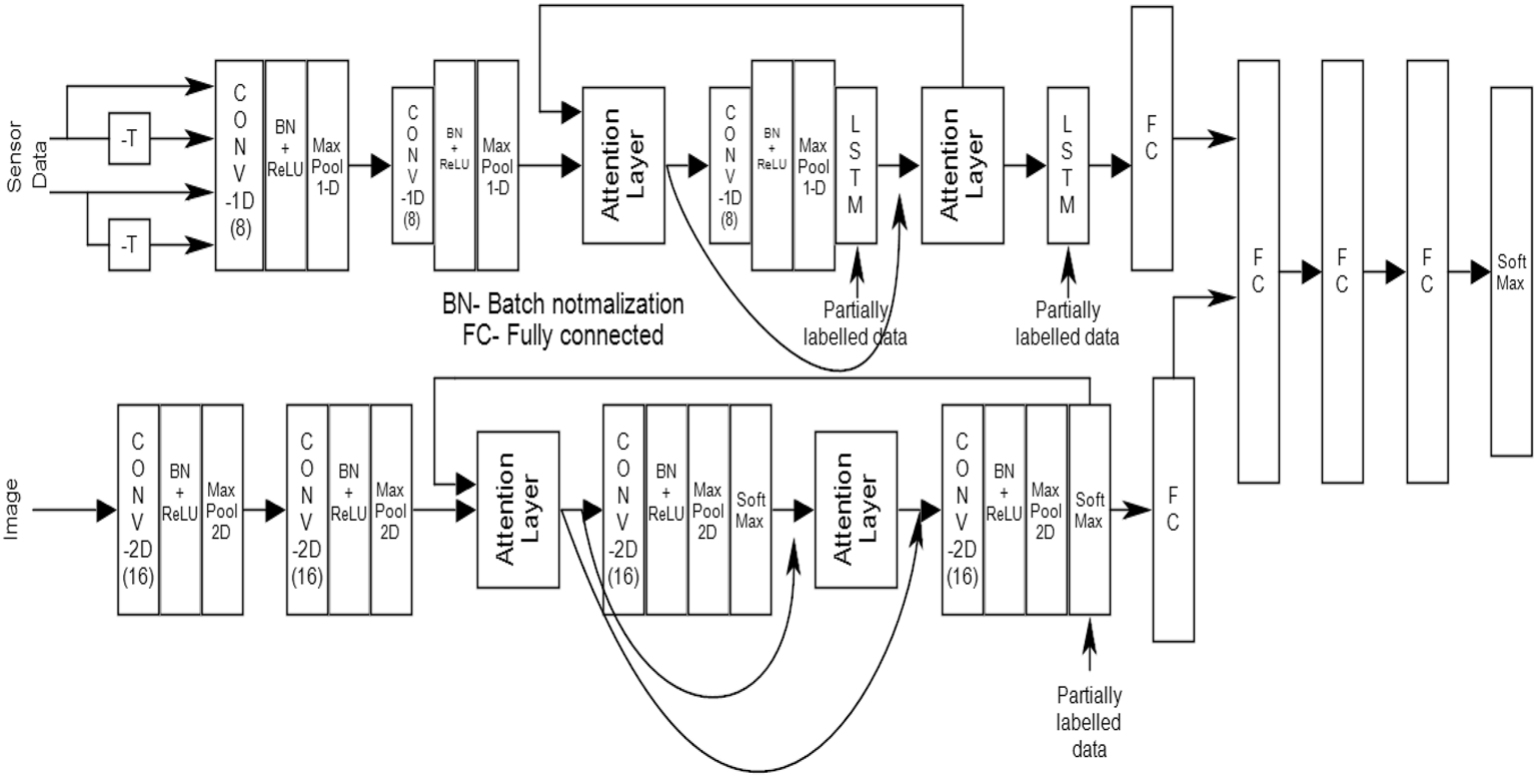
Cloud resident composite CNN + LSTM + attention mechanism for rainfall prediction and fruit health recognition.

**Figure 16:**
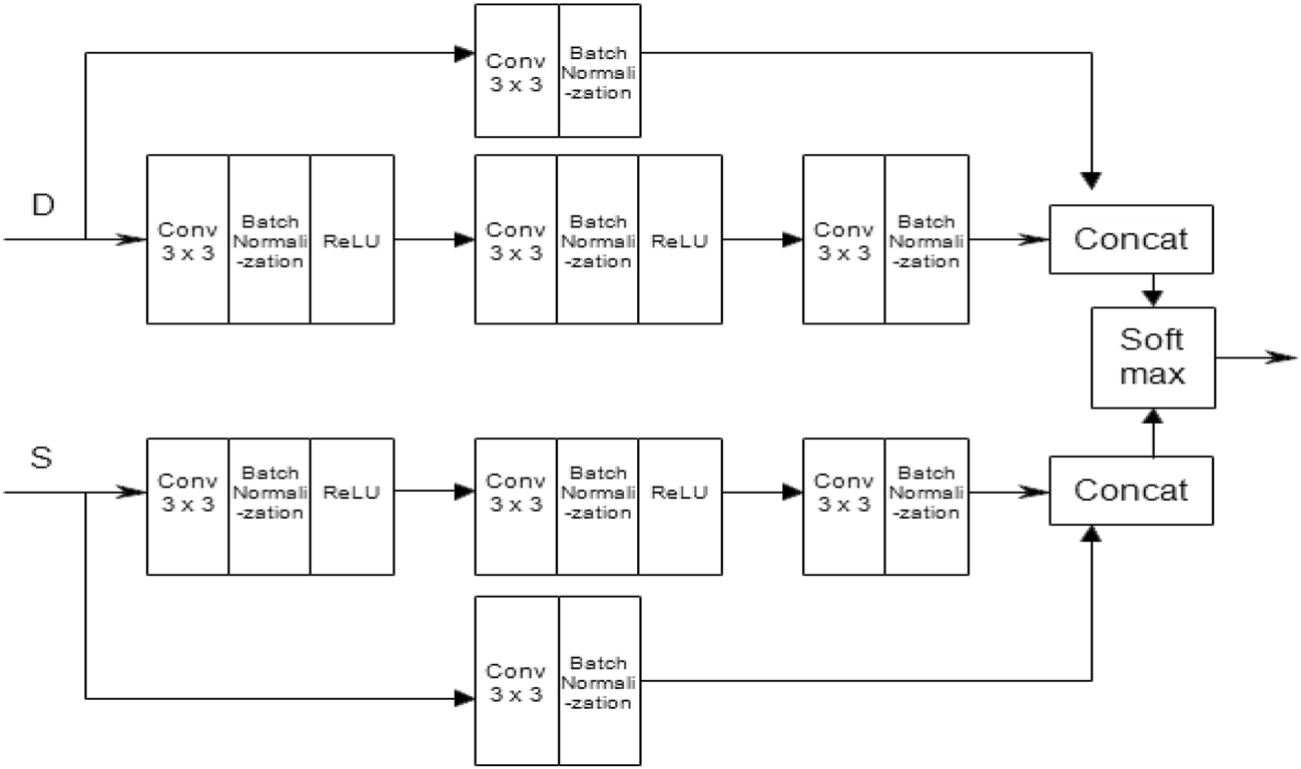
Multi head sectoral attention mechanism.

The above methods discussed provide the opportunity to explore the performance improvements that may be achieved by combining the CNN, LSTM and a multi head (MA) self-attention (SA) mechanism (Figs. 15, 16) in a composite framework (Fig. 15) for both forecast of rain and discrimination of fruit health. The mix of CNN, LSTM, and the SA mechanisms emerges out to be effective at discrimination tasks in a variety of areas^29–31^ that include natural language processing, computer vision, speech processing, etc. Let's look at how each part of the system contributes to the effectiveness of the whole framework.

The CNNs are utilized extensively for many tasks including the processing of images and texts. In the field of computer vision, CNNs are particularly useful at capturing hierarchical representations and small spatial patterns. They are made up of convolutional layers, which are followed by pooling layers, and they aid in the process of extracting useful characteristics from incoming data. CNNs are good at distinguishing between visual or sequential patterns, which makes them well-suited for tasks such as the classification of images and the identification of objects^21^.

The LSTM networks are a type of recurrent neural network (RNN) that perform well in the processing of sequential data, such as natural language sentences or time series. By combining a memory cell with input, forget, and output gates, LSTMs are able to accurately represent long-term dependence. This is made possible by the memory cell. Because of this architecture, LSTMs are able to remember information over time or forget it over time, which enables them to effectively handle sequential patterns that occur over a wide range of time scales. LSTMs are very helpful for activities such as analysing sentiments, translating languages, predicting time varying data and events, recognizing voices etc.^21^.

The SA mechanism, often referred to as the transformer architecture, has received a great deal of interest in recent years for its application in a variety of tasks that require special focus on specific areas of input patterns. The model is able to listen to different parts of the input sequence thanks to the self-attention capability, which also allows it to capture the dependencies that exist between words or tokens^21^. Calculating attention weights between all possible pairings of places in the input sequence, then computing a weighted sum of those attention weights to produce a context vector for each position, is what this function does. Because of this technique, the model is able to concentrate on the parts of the sequence that are the most important, which results in improved discrimination and an effective capturing of long-range relationships.

The combined model is able to take advantage of the benefits that are offered by each component namely CNN, LSTM, and Self-Attention processes. For instance, in an image classification challenge, the model might utilize the advantages of the CNN to extract local features and patterns from pattern embeddings, LSTMs to capture the sequential dependencies, and Self-Attention to attend to key sections of the sequence while taking into consideration the overall context of the problem. By utilising both the local and the global information that is contained within the input data, this combination enables the model to efficiently differentiate between the many groups or categories that are there.

**Figure 17:**
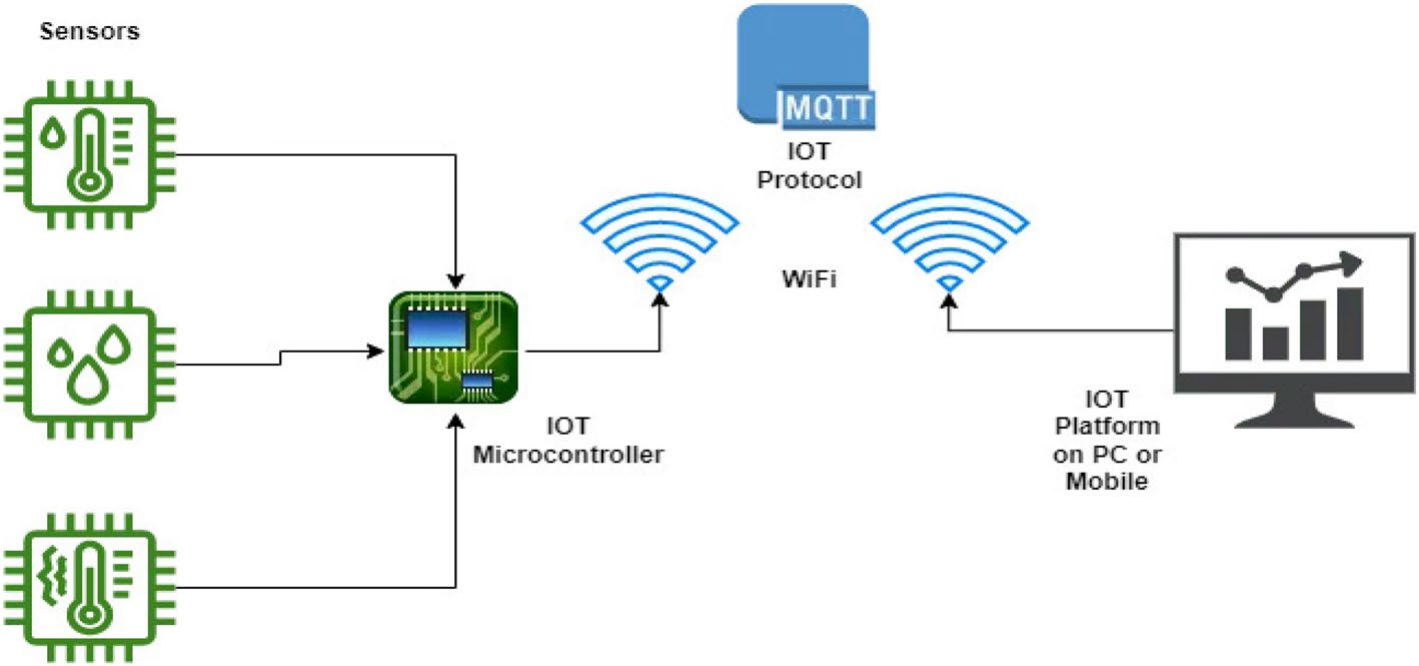
Architecture of the proposed agricultural management system.

As shown in Fig. 16, for the proposed approach has provision for using sensor data and image inputs. For the sensor data, there are actual and delayed feeds to retain the temporal context. Next, a block of 1D- convolutional (8 no.s) fed by 3 × 3 masks an dlinked to batach normalization (BN), rectified linear unit (ReLU) and max pooling (MPool) feeds another such block.It is followed by an attention layer (detailed layout Fig. 17) which drives another 1D conv + BN + ReLU + LSTM layers which dirve a MH-SA and a LSTM layer. This is followed by fully connected (FC) layers and ends at softmax block. For the image imputs, two blocks of 2D conv (16 with 3 × 3 masks) + BN + ReLU + MPool drives a FC layer which after a few more FC blocks ends in a softmax layer.

### Experimental set-up

The experimental set-up is formed by the IoT and ML methods as part of an agriculture farm management system (Fig. 17). The system is designed using NodeMCU and cloud computing and provides assistance to the farmers in monitoring soil moisture, air quality, humidity, and temperature as well as helps in predicting rainfall and fruit health conditions.

As already mentioned, the sensors are connected to the same NodeMCU platform (ESP 8266) and the data gathered from sensors are stored in a database in real-time. The ESP 8266 is connected to a nearby Wi-Fi network in order to establish a connection with the database's web server. The data can be downloaded from the real-time database and shown on the Android mobile app. MIT App Inventor is used to create the Android application. Depending on the user input through the pump controller, the app can use Boolean data (0 or 1) to convey the pump control status to the real-time database. The NodeMCU sends the command to the motor driver in order to regulate the irrigation process by retrieving the Boolean data from a real-time database. From Kaggle, a weather dataset is obtained and examined. In order to clean up the dataset and increase its value, the numerical, continuous, and categorical variables are separated, and each variable is then subjected to analysis. Following that, box-plot graphs are generated to exclude outliers and provide a visual representation of the probability distribution of the data.

Popular algorithms—CatBoost, Gaussian NB, and Random Forest Classifier and CNN + LSTM network—are used to forecast the best model for rainfall prediction after organizing the data and training. Further, a combined CNN + LSTM + Attention Layer based rainfall prediction and fruit health recognition system is designed which provides an integrated approach. The performance of this system is compared with the bench mark methods namely Cat-Boost, Gaussian NB, and Random Forest Classifier and CNN + LSTM network. Access to the web-server for these cloud resident tools is through an easy-to-use interface where the user may enter the criteria needed to predict when it will rain. Same is the case with fruit health monitoring.

## Results and discussion

In this section, we discuss the results derived and the related details.

### Performance of the IoT set-up

**Figure 18:**
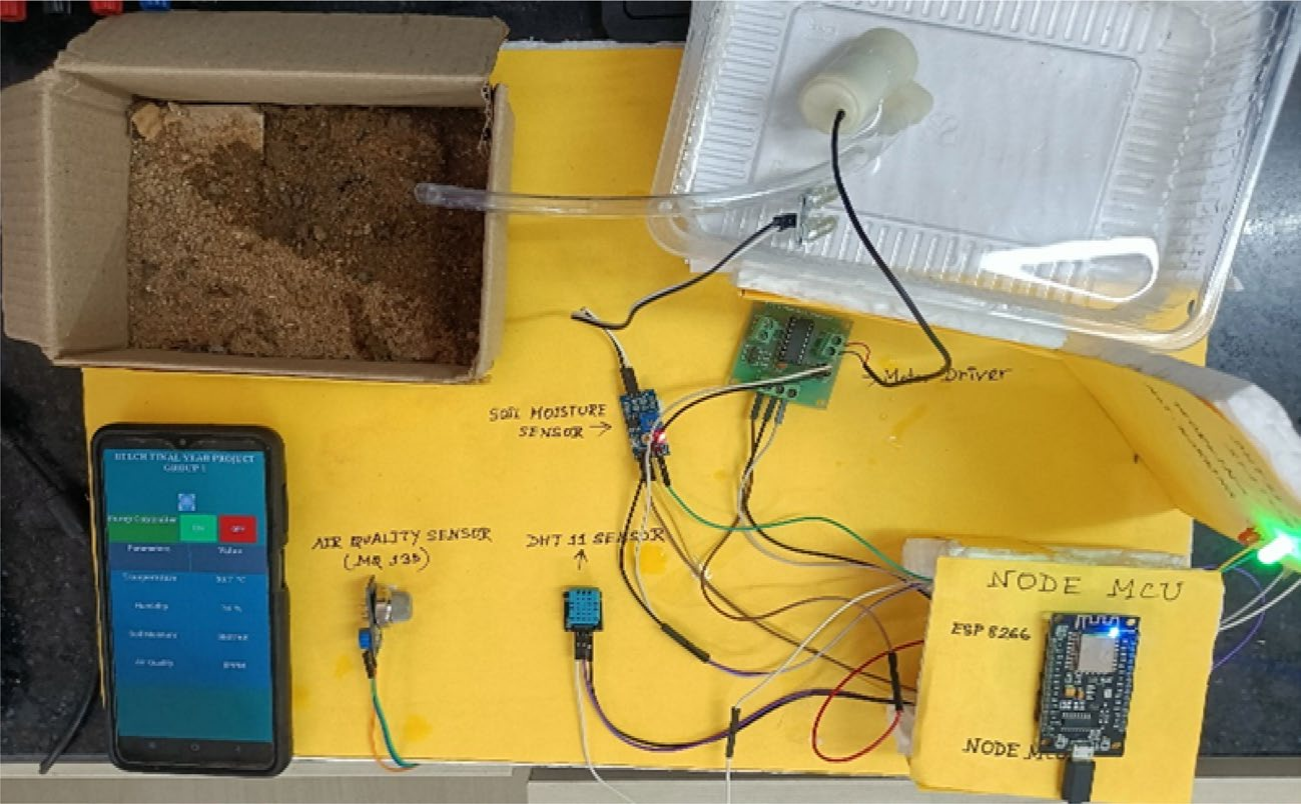
Experimental setup of the proposed IoT system.

**Figure 19:**
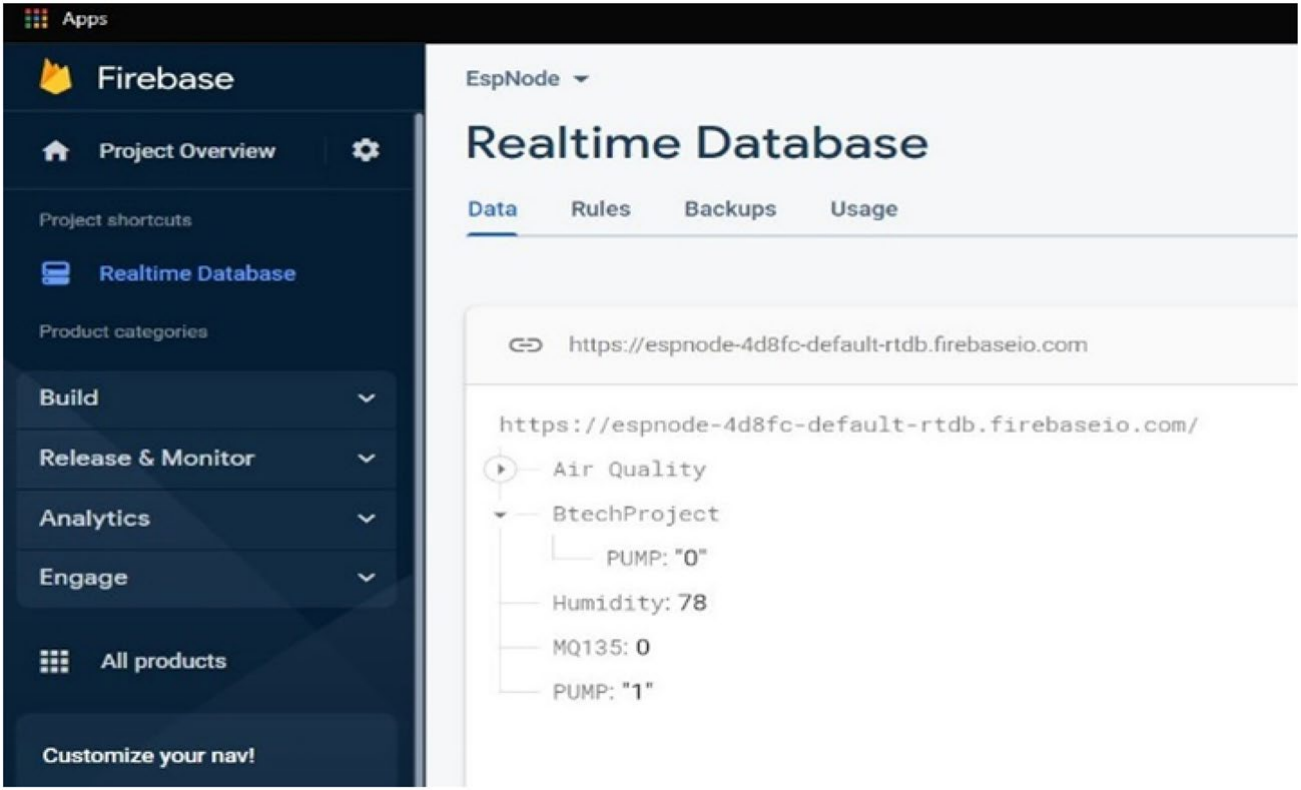
Web interface of the real-time Firebase database.

**Figure 20:**
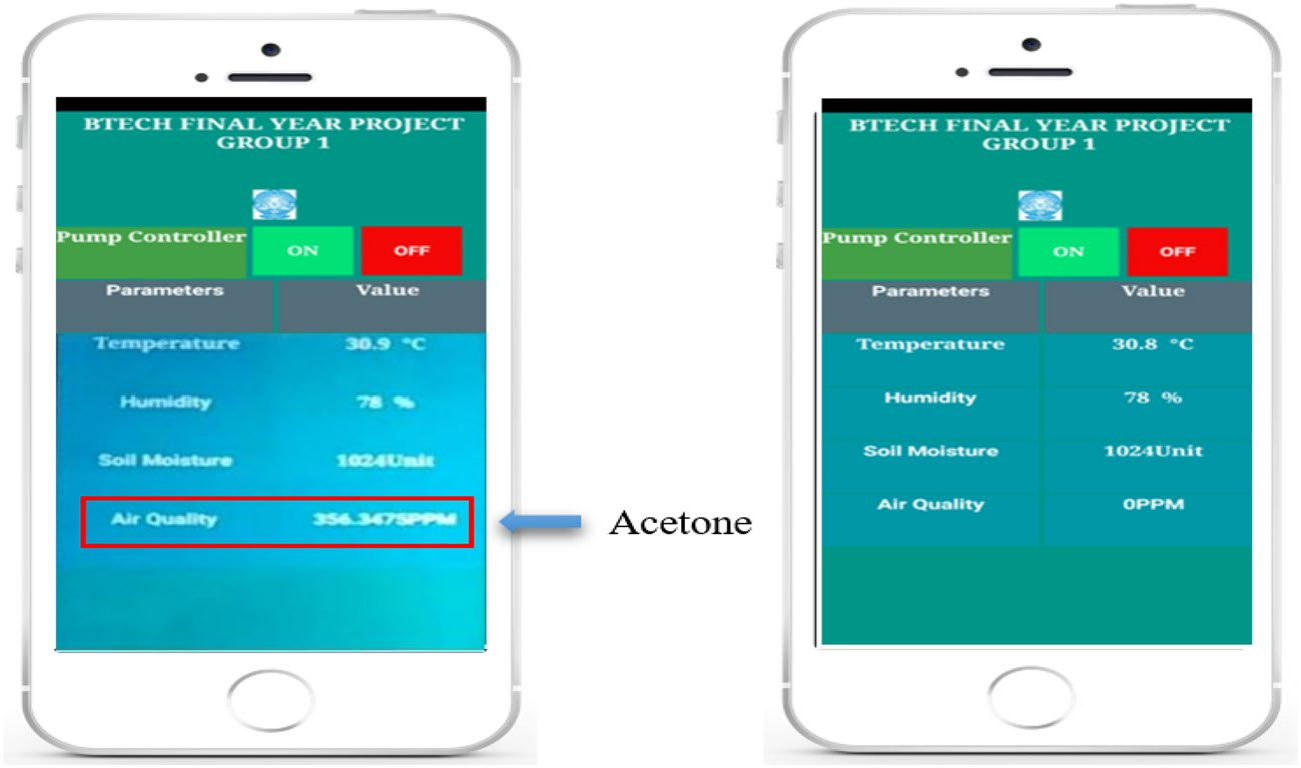
User interface of the Android application.

The primary programming code file is uploaded to the NodeMCU board using a USB cable after all hardware components have been installed. All findings are validated by looking at the serial monitor window. By manually disconnecting the DHT 11 sensor, the functionality of LED indicators is examined. It is confirmed that if the sensor is not connected to the microcontroller board, a red LED will light up, and a green LED will light up if all connections are sound. Figure 18 shows an experimental set-up of the system. Figures 19 and 20 show how all data are received and shown in the Android application monitor pane and Google Firebase real-time database. The water pump status is also checked by clicking ON/OFF button of pump controller in the Android application.

The sensors data are acquired and displayed in the mobile application. The monitor window shows the temperature, humidity, soil moisture and air quality (presence of acetone) information at two particular instants, in presence of acetone and in absence of acetone as shown in Fig. 19. Although acetone occurs naturally in the environment in plants, trees, volcanic gases, forest fires, the majority of the acetone released into the environment is of industrial origin. Acetone condensation products reported to have growth regulating properties for their effects on seed germination. It means that acetone measurement is crucial for agricultural lands nearer to the industrial areas. Table 3 shows sensor reading at certain reading instants.

**Table 3:** Sensors’ readings at different time instants.

Instants	Temperature (°C)	Humidity (%)	Soil moisture	Acetone (PPM)
1	30.8	78	1024	0.0
2	30.9	78	1024	356
3	22.0	68	971	373

User can also perform manual controlling of the motor through the mobile application based on the parameter measured. Table 4 shows certain expert inputs leading to the formulation of a decision logic derived out of the sensor readings.

**Table 4:** Parameters used in rainfall prediction model.

Soil Moisture		Pump status	Remarks
Value	Percent		
1024	100	Can’t start (“0”)	A value of 1024 means soil moisture is at saturation (very wet)
> 819.2	> 80	OFF (“0”)	Values above 80% indicate excess moisture stress
552.9	54	OFF (“0”)	A value of 54% means near optimum soil moisture for plant growth
< 204.8	< 20	ON (“1”)	Values below 20% indicate drought
0.0	0.0	ON (“1”)	A value of 0 means soil moisture is at the wilting point (very dry)

### Rainfall prediction

A few algorithms are implemented and their AUC as well as accuracy score are checked.

#### CatBoost

**Figure 21:**
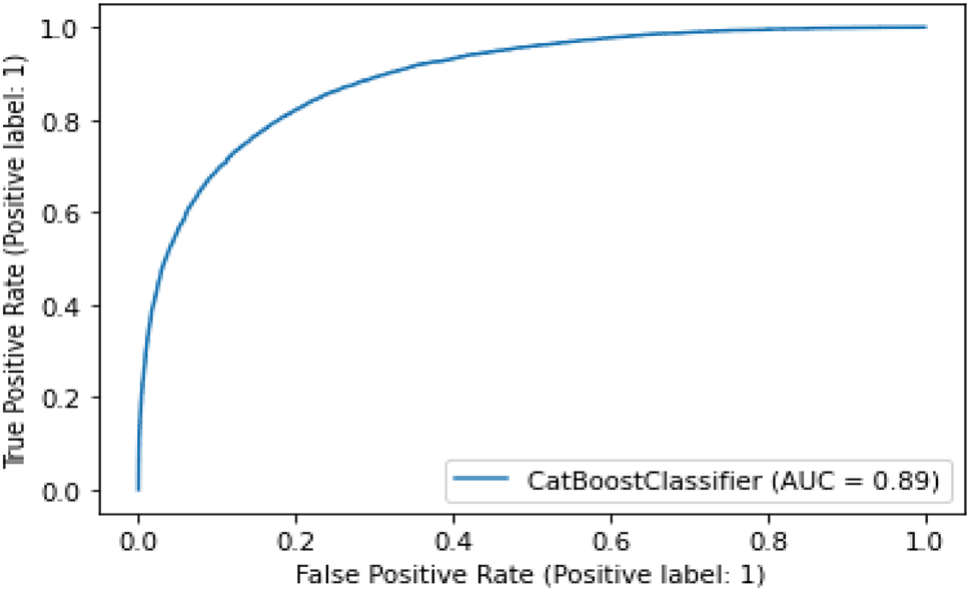
Experimental result of CatBoost classifier.

The following algorithm suits the model perfectly as because it gives an accuracy score of 0.86 and AUC score of 0.89 as shown in Fig. 21.

#### Random forest

**Figure 22:**
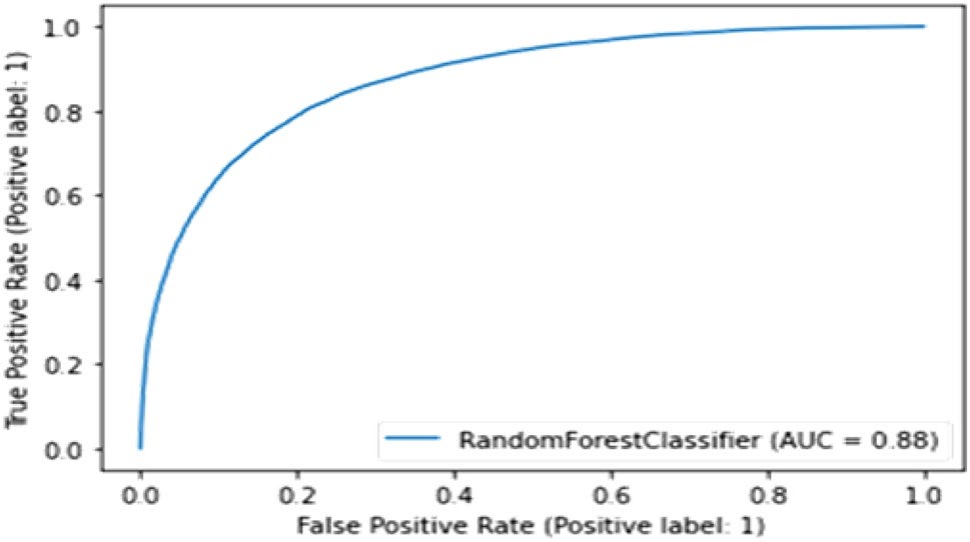
Experimental result of Random Forest classifier.

This algorithm suits the model but it has less accuracy and AUC score than CatBoost algorithm but it can also predict rainfall pretty well as shown in Fig. 22.

#### Gaussian NB

**Figure 23:**
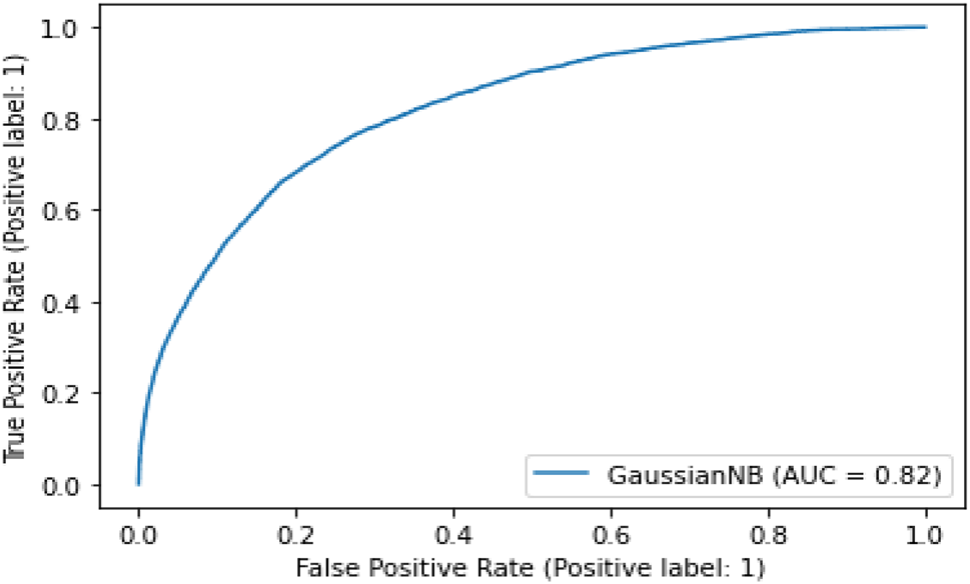
Experimental result of Gaussian NB classifier.

This is the least accurate model as both the AUC as well as the accuracy score are less than both CatBoost and Random Forest classifier as shown in Fig. 23.

**Figure 24:**
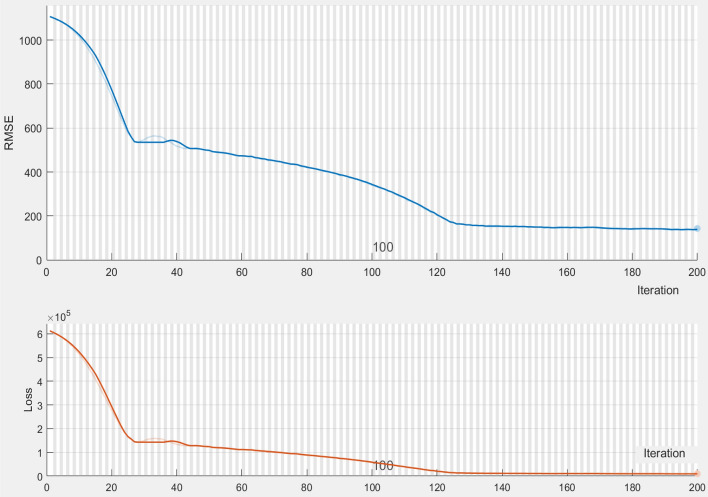
Root mean square and loss function values of the CNN + LSTM model used for rainfall prediction.

Similarly, the CNN in the VGG16 configuration^21^ with LSTM layer at the end is trained with sensor data of two weeks and validated (Fig. 24). All the data are analyzed and summary outlined in Table 5.

**Table 5:** Summary results representing the AUC and accuracy score derived for rainfall prediction.

Algorithms	AUC Score	Accuracy (%)	Remarks
Cat-boost classifier	0.89	86	Better model
Random forest classifier	0.88	84	Satisfactory
Gaussian NB classifier	0.82	75	Least score
CNN + LSTM	0.92	94	Most suitable

As shown in Table 5, among the different prediction techniques, the CNN + LSTM and Cat-Boost yield the best accuracies. However, CatBoost classifier has a higher memory usage and performs better when categorical data is available. Further, random forest classifier has greater processing criticality and requires long training times. Similarly, the Gaussian NB classifier assumes feature independence, an unrealistic consideration in case of real-world data. Moreover, the Gaussian NB classifier encounters the zero-probability situation which provides erroneous outcomes. Therefore, the CNN + LSTM combination is selected for the predicting the rainfall.

### Rainfall prediction

**Figure 25:**
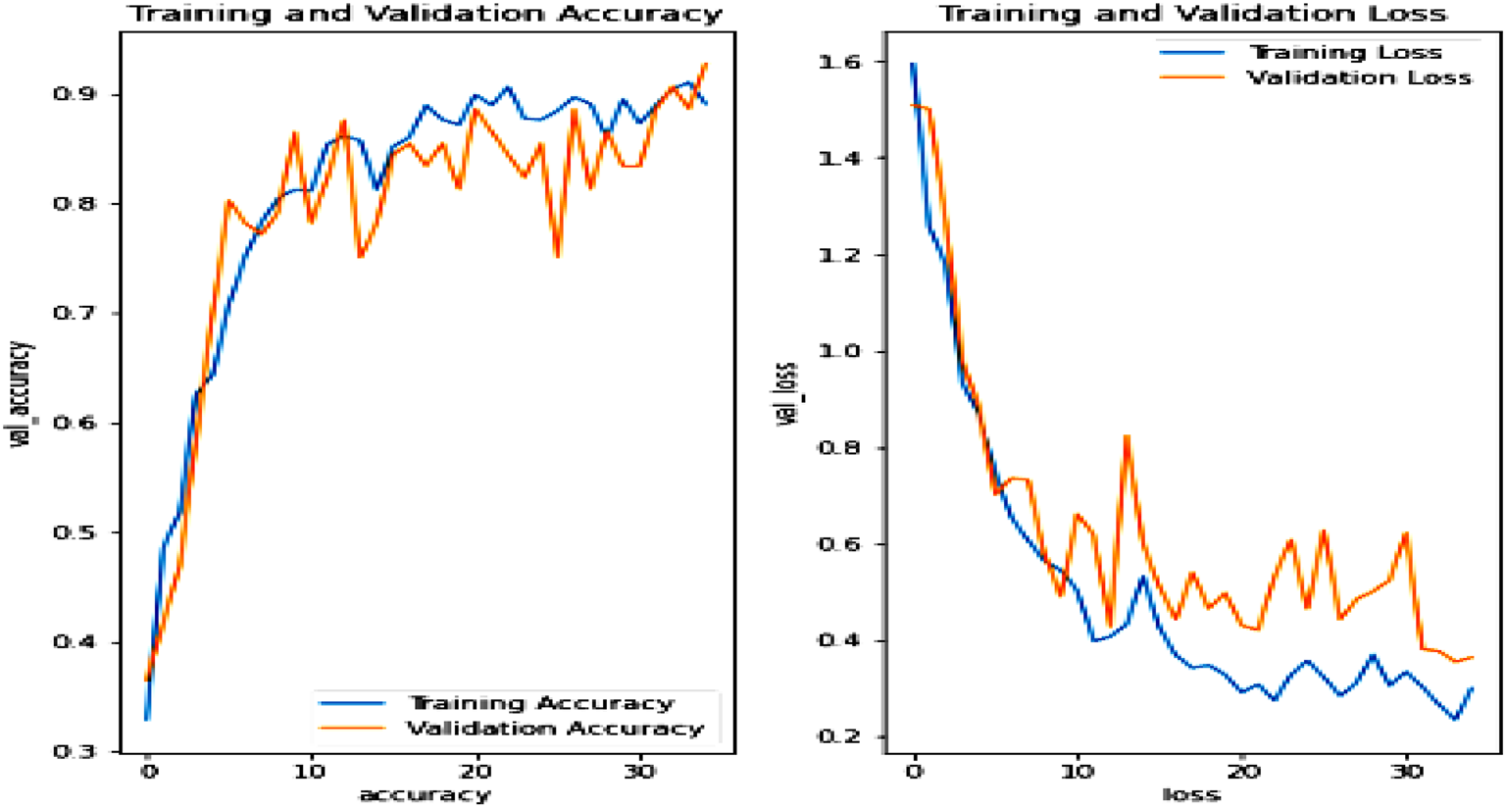
Experimental result of CNN + Softmax classifier.

**Figure 26:**
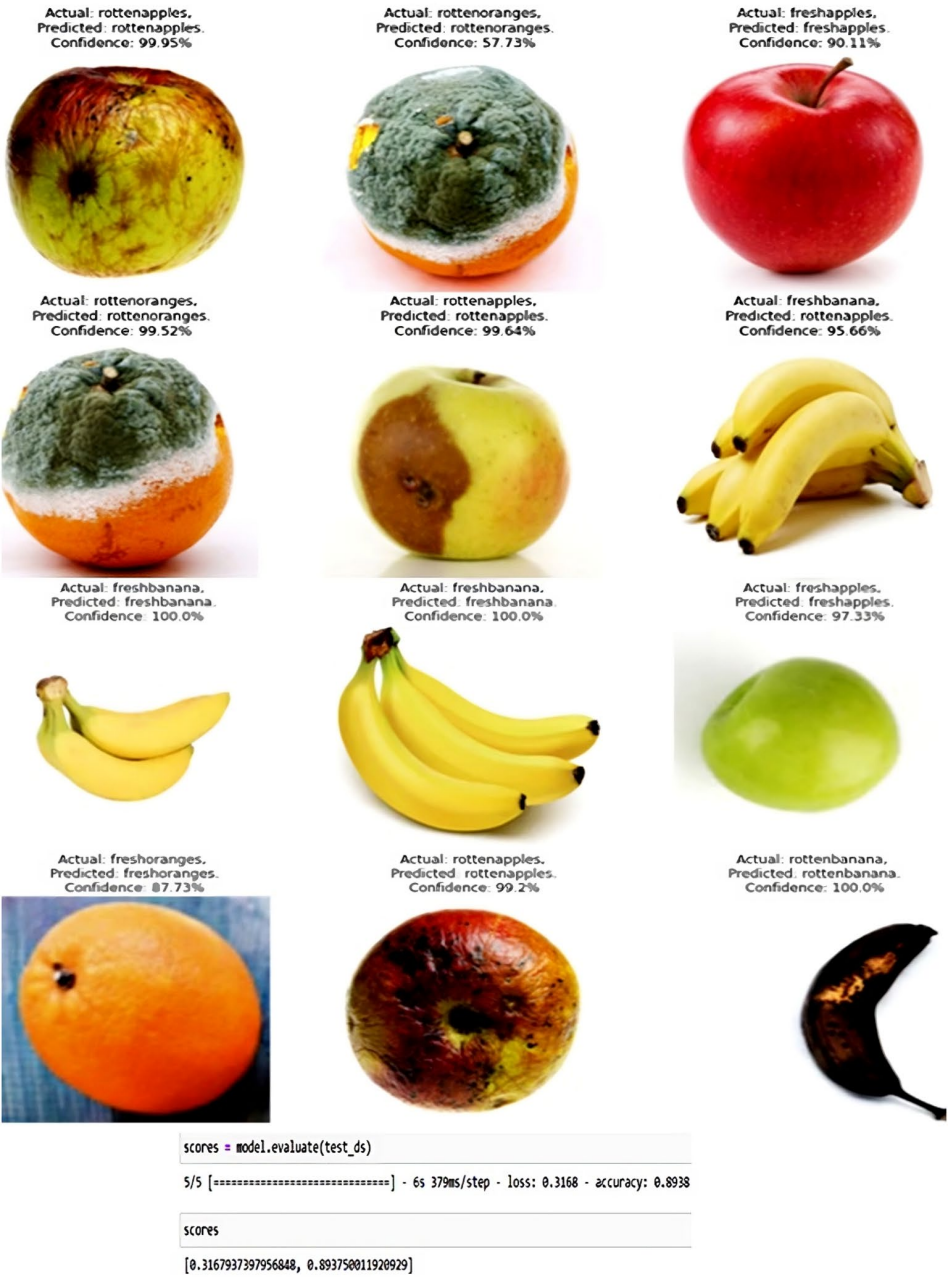
Experimental results of fruit health recognition model.

After data augmentation, the graph is plotted featuring training and validation accuracy and training and validation loss. The blue line in Fig. 25 represents the training part and orange line represent validation part. The worst case (trained with a data set and tested with a sequence with illumination and resolution variation) accuracy is found out to be 89.38% and loss score is found out to be 0.3168 as shown in Fig. 25. The experimental results are shown in Fig. 26 obtained from the CNN + Softmax combination. Table 6 featuring the actual and the predicted identities along with confidence percentage obtained using a CNN classifier shows the reliability of the approach. Similar set of results are obtained for the other classifiers as well (CNN with Adam Optimizer, ResNet50 and GoogLeNet). This is shown in Table 7.

**Table 6:** Truncated depiction of actual and predicted output of the fruit health recognition model for different fruit samples.

Fruit Samples	Actual fruit	Recognized fruit	Confidence (%)
Apple 1	Rotten apple	Rotten apple	99.95
Orange 1	Rotten orange	Rotten orange	57.73
Apple 2	Fresh apple	Fresh apple	90.11
Orange 2	Rotten orange	Rotten orange	99.52
Apple 3	Rotten apple	Rotten apple	99.64
Banana 1	Fresh banana	Rotten apple	95.66
Banana 4	Fresh banana	Fresh banana	100.0
Banana 5	Fresh banana	Fresh banana	100.0
Apple 5	Fresh apple	Fresh apple	97.33
Orange 3	Fresh orange	Fresh orange	87.73
Apple 6	Rotten apple	Rotten apple	99.20
Banana 6	Rotten banana	Rotten banana	100.0

**Table 7:** Performance of different classifiers as part of the fruit health recognition system.

Sl. No	Method	Accuracies (%)
1	CNN + Softmax	93
2	CNN with adam optimizer	94
3	ResNet50	91
4	GoogLeNet	90

### Interface of the websites

**Figure 27:**
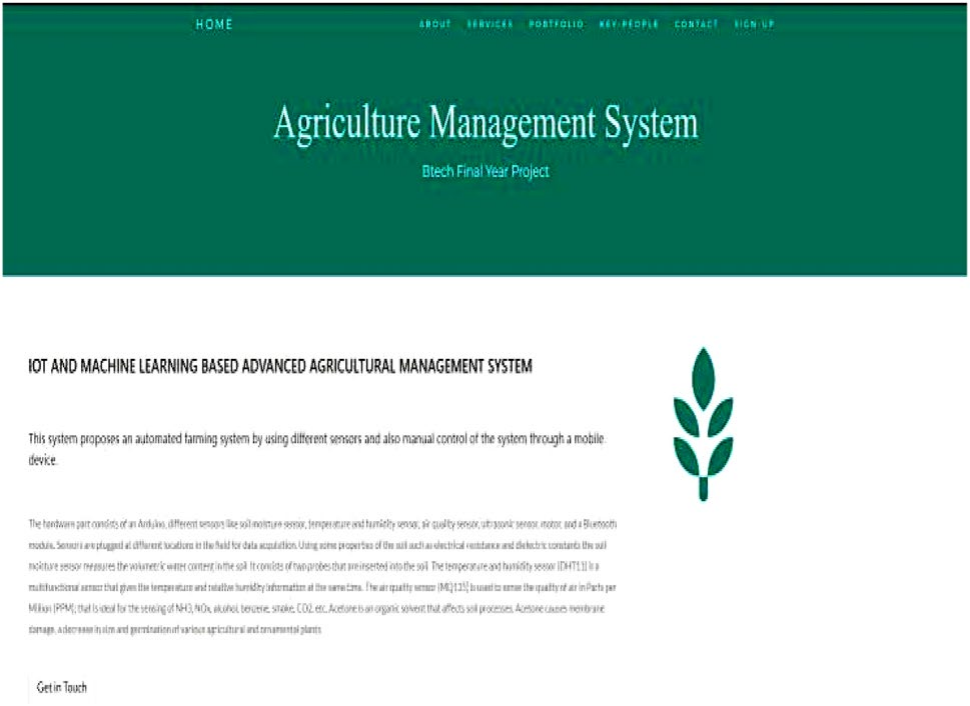
Agricultural Management System website home page.

**Figure 28:**
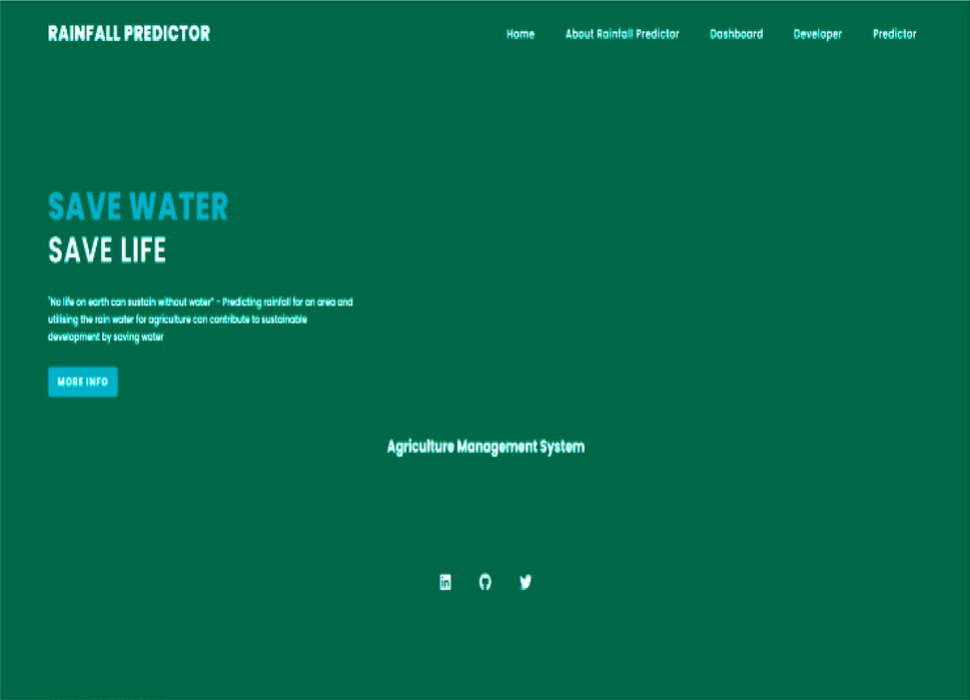
Home page of fruit recognition website.

A cloud-based service has been made available for online testing. Figures 27 and 28 show the screenshots of the web-interfaces. Cultivators of different parts of the north-east Indian state of Assam have been invited to use the services of the cloud resident app. The cultivators experienced latency in responses which varied with time and location. It is dependent on wireless coverage. But the accuracy obtained is as reported above. The impact of the system is associated with the fact that the buyer–seller relationship can be reinforced with the help of the system which has been made available for testing using the web-interface.

### Comparison with previous work and discussion

A description of certain statistical indicators for predicting rainfall, identifying fruit health, and comparing current results is shown in Table 8. Additionally, it covers the memory requirements for each of the techniques applied in the work. We have taken into account categorical (17), continuous (14), discrete (2), and numerical (16) characteristics. Integers and floats are represented using numerical characteristics. Only one value can be used for discrete characteristics. There is a range of values for continuous features. Levels, or values, can be assigned to categorical features. In case of rainfall prediction, even though the CNN + LSTM combination requires more memory (than Gaussian NB Classifier) but lower than Cat-Boost Classifier, Random Forest Classifier, it performs better because due to the presence of the LSTM the tracking of the time dependent samples becomes superior. Even compared to CNN, the Adam Optimizer's support to CNN provides excellent accuracy and is supported by memory-efficient processing. Systems with low memory requirements are more useful when running cloud-based apps. However, they are not appropriate for use in transfer learning. The hybrid *CNN* + *LSTM* + *MHSA* combination provides better accuracy, is light weight (as optimization is achieved), takes lesser training and testing times. Hence, it is found to be more suitable for a cloud resident combined rainfall predictor and fruit health recognizer. Exactly identical works have not been reported but a work of similar nature is^32^ where the authors have used ML and DL methods for winter wheat yield prediction using an extensive dataset of weather, soil, and crop phenology variables reporting an efficiency of around 90%. The key aspect of the present work is its better precision (97%) while it combines fruit health monitoring and rainfall prediction using certain sensor inputs and samples images.

**Table 8:** Summary of certain statistical metrics of rainfall prediction and fruit health recognition and comparison with previous results.

Sl no	Classifier	Application	Memory used in MB	Precision	Recall	F-measure	Training time (s)	Testing time (s)
1	Cat-boost classifier	Rainfall prediction	2390	0.81	0.88	0.84	5371	389
2	Random forest classifier		2390	0.79	0.83	0.81	5234	440
3	Gaussian NB classifier		0.23	0.78	0.55	0.64	5549	489
4	CNN + LSTM		550	0.94	0.93	0.95	5608	140
5	^19^		–	0.78	0.83	0.80	6520	530
6	CNN + Softmax	Fruit health recognition	550	0.93	0.93	0.93	6500	330
7	CNN with adam optimizer		500	0.94	0.94	0.94	3279	220
8	^20^		–	0.97	0.97	0.96	6650	440
9	ResNet50		105	0.91	0.90	0.92	5101	122
10	GoogLeNet		100	0.90	0.91	0.91	5109	129
11	CNN + LSTM + MHSA	Rainfall prediction and fruit health recognition	375	0.97	0.96	0.97	2430	114

Its better performance in certain situations is due, in part, to the unique capabilities brought to the design by combining CNN with LSTM, MHSA, and softmax layers. The LSTM layers capture dependencies and patterns in sequential data, but CNN excels in capturing spatial hierarchies of features in pictures or sequences and recognizing patterns independent of their location in the input space. Capturing input sequences' global dependencies is an area where the MHSA performs well. Down this way, the model might have focused on pertinent data and create long-range relationships by giving varying weights to various portions of the input sequence. Rainfall prediction and fruit health monitoring have both benefited from these improvements while adopting sensor data and picture processing.

Here, we've described how to create a NodeMCU-based network, which offers benefits like easy database linkage, built-in WiFi connectivity, and good communication range. This technology supports a range of a few hundreds of meters and may be utilized both indoors and outdoors. The platform delivers a fusion of the sensors when every sensor is linked to NodeMCU. It is feasible to locate and identify sensor connection issues. A motor controller and real-time data monitoring for data analysis are included with the mobile app. The motor controller controls the condition of the water pump. The website is enhanced with all of the tools used for fruit health assessment and rainfall forecasting. The fruit health recognition webpage offers a good analysis of the input photographs to identify whether the fruit is rotting or ripe, and the rainfall prediction forecasts the weather for the following day. Data on soil moisture and temperature are collected with accuracy and precision by the modern agriculture management system developed utilizing IoT and ML techniques. Additionally, it helps farmers increase agricultural productivity and provide an atmosphere that is effective for growing food. It contributes to a decrease in over- and under-irrigation, soil erosion, and water waste by providing data from the used sensors. So, an automated system for monitoring fruit health and providing farming help with relation to water resources is a crucial tool for the cultivator and entrepreneurs. The cost-effectiveness of the whole effort can be improved with an automated method. In addition to the foregoing, the offered methodologies have quick execution times and dependable decision-making, which cannot always be matched by employees hired for the job. The sensor pack offers exact cutoff points at which water sprinklers could start. Additionally, it aids in reducing water waste. With human intervention, it would not always be possible to maintain the precise timing and volume of watering. With regard to fruit health monitoring, the same is true. The laborious and thorough nature of the work done by human workers may be inaccurate. A scalable, profitable, and effective production system can be developed using an IoT and machine learning (ML)-based agriculture management system. The statistical metrics for predicting rainfall, identifying fruit health, and comparing current results are shown in Table 8. The benefits of the suggested approach are obvious. However, the performance of the system is dependent on the latency of response which is a factor determined by wireless network coverage and certain hardware dependence.

## Conclusions

Here, we reported the design of a precision agriculture setup with stress on adoption of sensors, IoT and ML to regulate water flow and process management so as to maximize crop yield. The work deals with the workings of various sensors, including soil moisture sensors, temperature and humidity sensors, and air quality sensors, in an IoT package to collect and monitor the real-time status of an agriculture management system associated with rainfall prediction and fruit health monitoring. The key aspect of the work is the design and configuration of a few ML algorithms namely CatBoost, Gaussian NB, Random Forest and CNN + LSTM classifiers trained to provide accurate rainfall prediction, fruit health monitoring and recognition (carried out using CNN + Softmax, Adam optimizer, GoogLeNet and ResNet50) and provide area under curve (AUC) scores as well as accuracy value under on field conditions. Further, we have designed a combined rainfall predictor and fruit health recognizer using a CNN + LSTM and a multi-head self-attention mechanism. This approach requires lesser memory, is more reliable and requires low computation time. These provide sufficient ground for formulation of an integrated agriculture assistant for cultivation of certain types of fruits. Experimental results show that the approach is precise and robust and the services are hosted using a web portal with a user-friendly interface.

## Data Availability

When requested, the authors will make available all data used in this study. Please, contact Prof. Kandarpa Kumar Sarma (kandarpaks@gauhati.ac.in). **Software Used**: For all our activities we used the following software. 1. Matlab 2020 version (available from www.mathworks.com), 2. Google Colab (available from www.google.com), Blynk app (available from https://blynk.io/). We have legal accesses and all licenses to use these software products.
